# Supplementary figures and images for: Expansion of RiPP biosynthetic space through integration of pan-genomics and machine learning uncovers a novel class of lanthipeptides
Source: PLoS Biol. 2020 Dec 22;18(12):e3001026. doi: 10.1371/journal.pbio.3001026 (PMC7794033; doi:10.1371/journal.pbio.3001026)

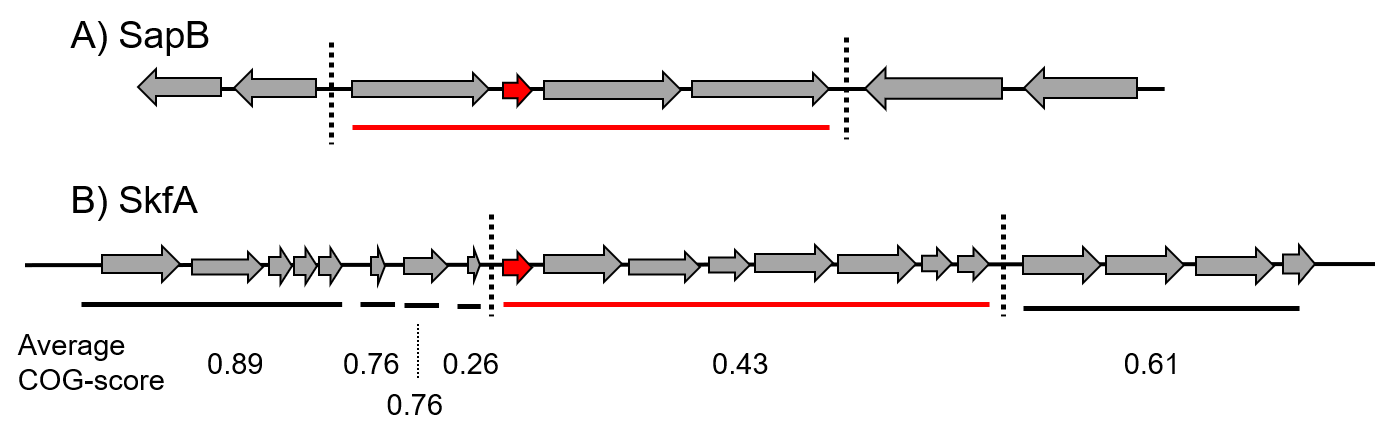

Supplement: S1 Fig — Two examples are provided here to illustrate identification of putative gene clusters in decRiPPter. (A) In the sapB gene cluster, 4 genes form the main BGC. These 4 genes are sequential, share the same strand orientation, and lie within a small distance of one another (< = 50 nt). They are therefore fused together into a single gene cluster. The flanking genes are on opposite strands and therefore not considered. (B) The skfA BGC consists of 8 genes sequential genes that share the same strand orientation. However, it is flanked by several other genes that also share the same strand orientation, within relatively short intergenic distances (< = 200 nucleotides). Using the island method, the genes are first fused into 6 islands, within 50 nucleotides distance of one another (indicated by lines underneath the genes). These islands may then be fused depending on the COG-score, which does not happen here because the difference is too large. The result is that the flanking genes, with a too high COG-score, are not added, and the correct BGC remains. BGC, biosynthetic gene cluster; COG, cluster of orthologous genes; decRiPPter, Data-driven Exploratory Class-independent RiPP TrackER. (PNG) [file pbio.3001026.s001.png]

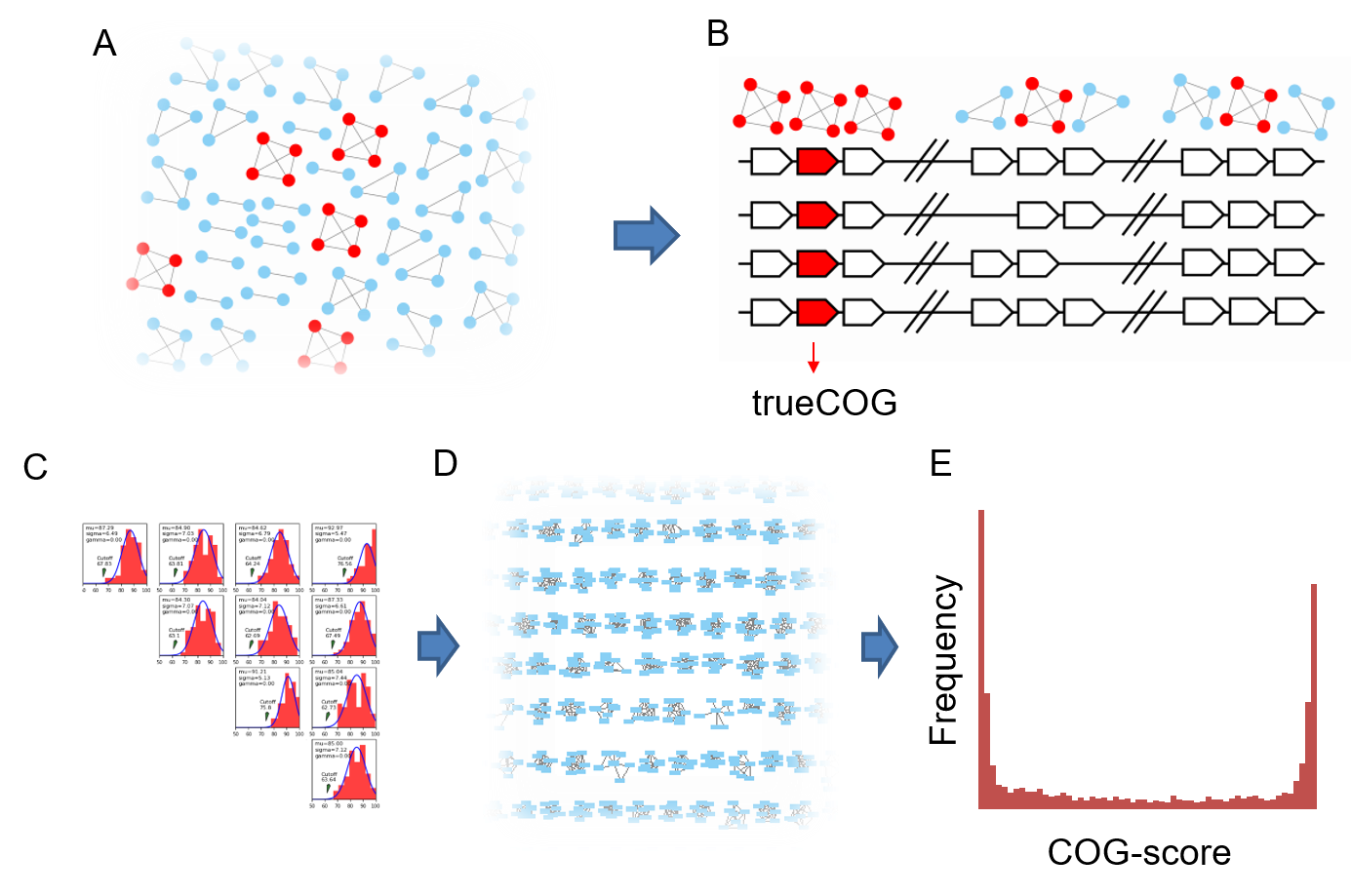

Supplement: S2 Fig — In this example, the COG scores of 4 genomes are calculated. (A) All encoded proteins are aligned to find BBHs (edges). All clusters of BBHs conserved across all genomes are displayed as red. If 1 genome does not contain a homologous gene, or the gene in question is not a BBH with all genes from the cluster from other genomes, it is not considered a conserved group of BBHs. (B) If the flanking genes of the clusters of BBHs are also part of clusters of BBHs, the center genes are considered to form a trueCOG. Of the 3 cases displayed here, only the leftmost group passes this criterion; for the center group, not all genes are conserved; and for the right group, not all genes are BBHs with one another in the flanking groups. (C) The genes in all trueCOGs between each genome pair are used to create a sequence identity cutoff to use for all protein-coding genes in a given pair of genomes. (D) All genes are paired using the sequence identity cutoffs determined in the previous step. (E) The COG-score is calculated for each gene. Typically, a bimodal distribution can be seen, with many genes either conserved across all genomes, or only present in a single organism. BBH, bidirectional best hit; COG, cluster of orthologous genes; decRiPPter, Data-driven Exploratory Class-independent RiPP TrackER; trueCOG, true cluster of orthologous genes. (PNG) [file pbio.3001026.s002.png]

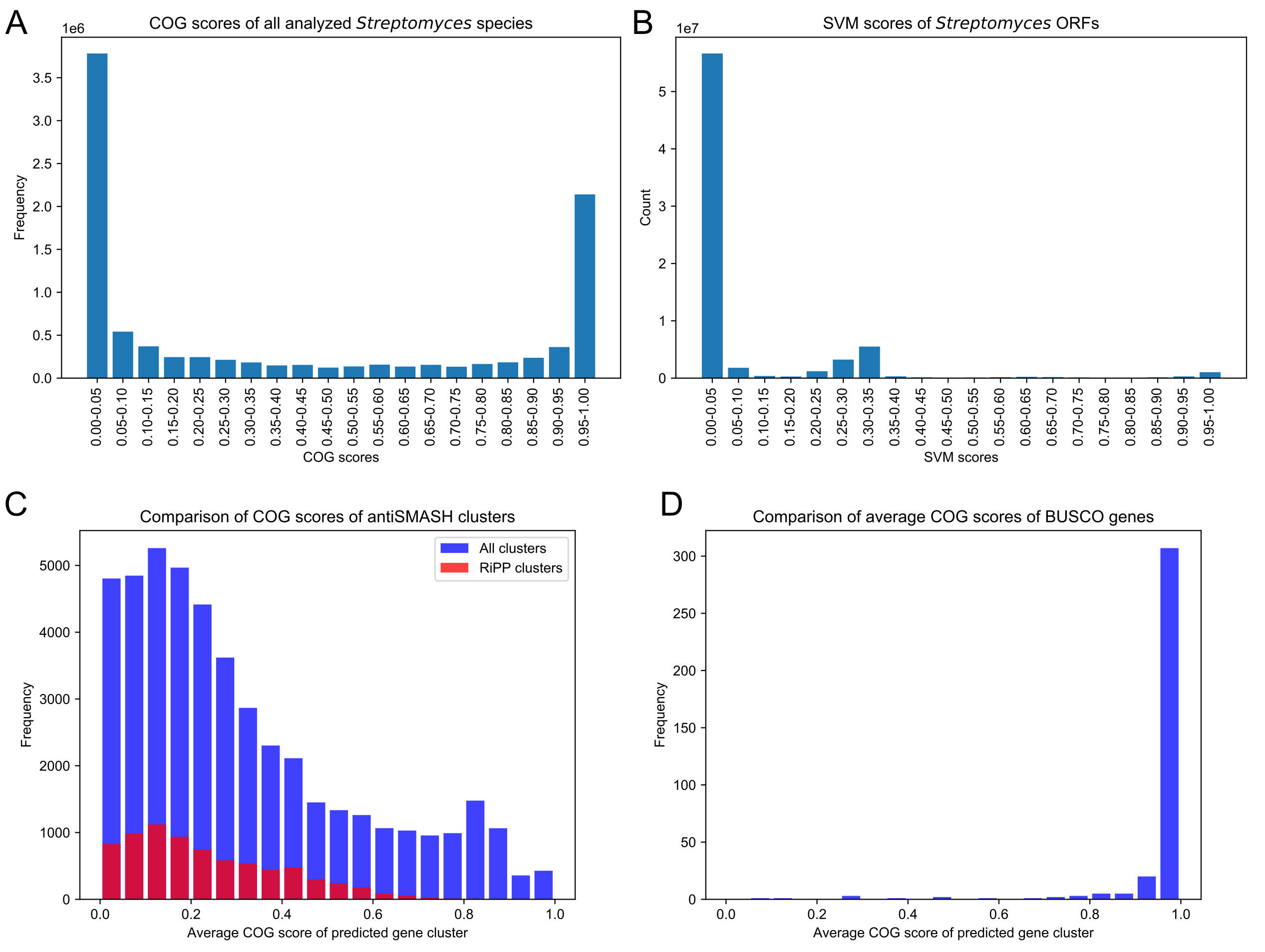

Supplement: S3 Fig — (A) COG scores of all genes in all 1,295 analyzed Streptomyces genomes. A high COG score indicates presence of homologs in many different genomes, while a low COG score indicates a more infrequent distribution. COG scores were calculated as described in the methods. (B) Distribution of the scores assigned by decRiPPter’s SVM classifier. A total of 7.1 * 107 small ORFs were analyzed. Based on the slight enrichment of precursors with scores > = 0.90, the cutoff was set at 0.9. (C) Comparison of COG scores of antiSMASH-detected gene clusters. COG scores were averaged over all genes in the predicted gene clusters. COG scores averaged 0.311 ± 0.249 for all gene clusters, and 0.234 ± 0.166 for RiPP gene clusters. (D) Comparison of average COG scores of BUSCO genes. The average of each BUSCO [51,52] gene was calculated for each genome analyzed. Numerical data of figures A, B, C, and D are available in S3 Data. BUSCO, Benchmarking set of Universal Single-Copy Orthologs; COG, cluster of orthologous genes; ORF, open reading frame; RiPP, ribosomally synthesized and post-translationally modified peptide; SVM, Support Vector Machine. (PNG) [file pbio.3001026.s003.png]

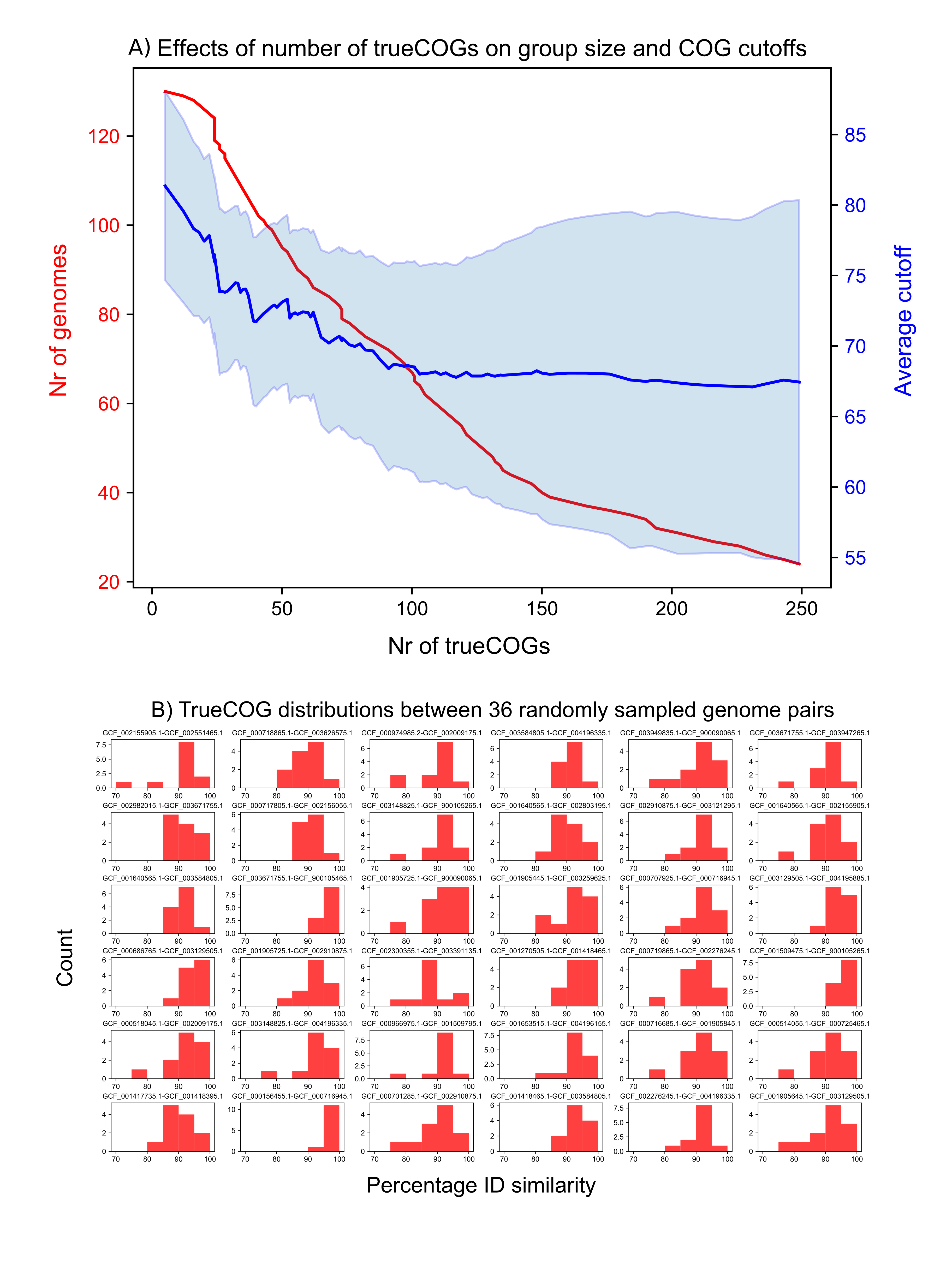

Supplement: S4 Fig — (A) As the minimum number of trueCOGs increases, the number of genomes that can be analyzed together (red line) decreases. In addition, the average COG cutoff (blue line) decreases when more trueCOGs are added, and the spread of COG cutoffs (shaded area; average cutoff ± the standard deviation) increases, suggesting that additional trueCOGs that were added were less conserved and showed higher variability in sequence similarity. (B) TrueCOG distribution between 36 randomly sampled genome pairs. Based on these distributions, COG cutoffs were determined. Numerical data of figures A and B are available in S3 Data. COG, cluster of orthologous genes; trueCOG, true cluster of orthologous genes. (PNG) [file pbio.3001026.s004.png]

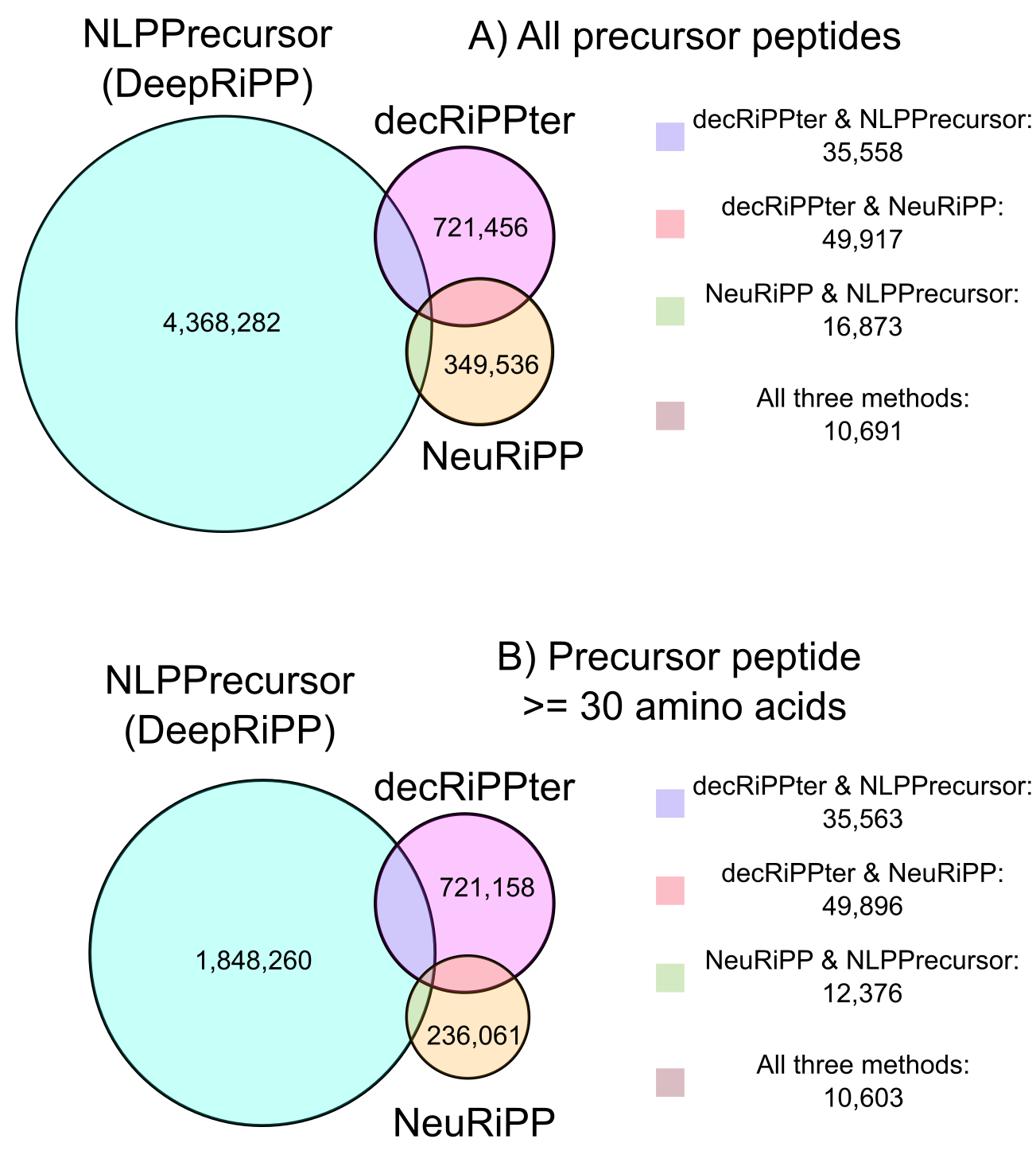

Supplement: S5 Fig — All small ORFs from the 1,295 Streptomyces genomes were classified by DeepRiPP’s NLPPrecursor [35] module, NeuRiPP [34], and decRiPPter. The 3 tools have only a small overlap (10,691 hits). NLPPrecursor scored 6 times more hits as positive, and NeuRiPP roughly half when compared to decRiPPter. Many of these hits were very small ORFs (≤30 amino acids; (B)), though, while most of decRiPPters predicted precursors were larger than that. The exact accuracy of these tools cannot be determined, as it is unclear which of these hits are false positives, and which are hits in novel RiPP BGCs. BGC, biosynthetic gene cluster; ORF, open reading frame; RiPP, ribosomally synthesized and post-translationally modified peptide. (PNG) [file pbio.3001026.s005.png]

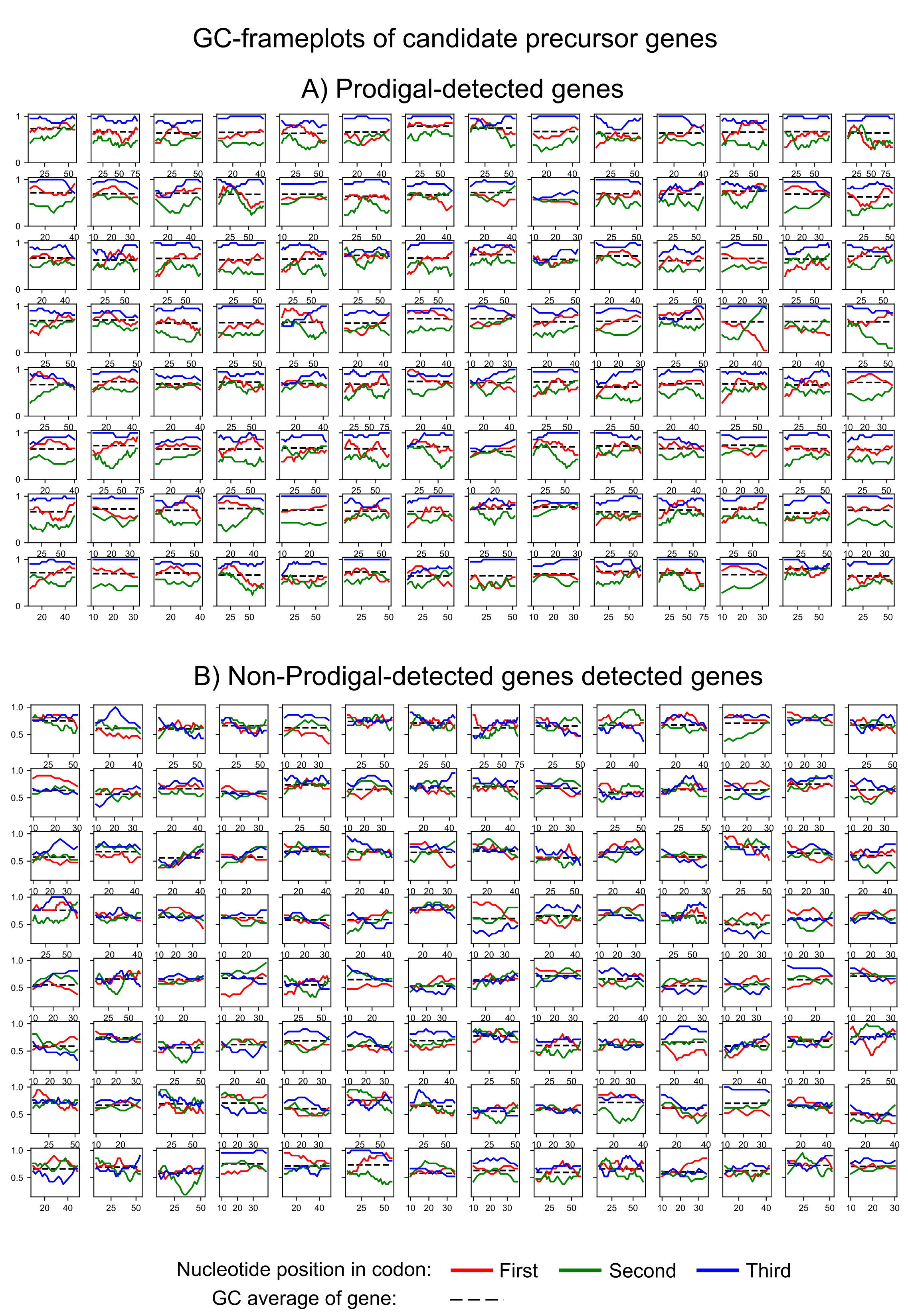

Supplement: S6 Fig — GC-content of randomly sampled Prodigal-detected precursor hits (A) and intergenic precursor hits (B). GC content is shown as the moving average of the first, second, and third positions, using a window-size of 5 and a step-size of 2. Only a small percentage of intergenic hits showed clear distinction between the 3 moving averages as in the Prodigal-detected hits, suggesting the majority of these are not encoding genes. Numerical data of figures A and B are available in S3 Data. GC, guanine-cytosine. (PNG) [file pbio.3001026.s006.png]

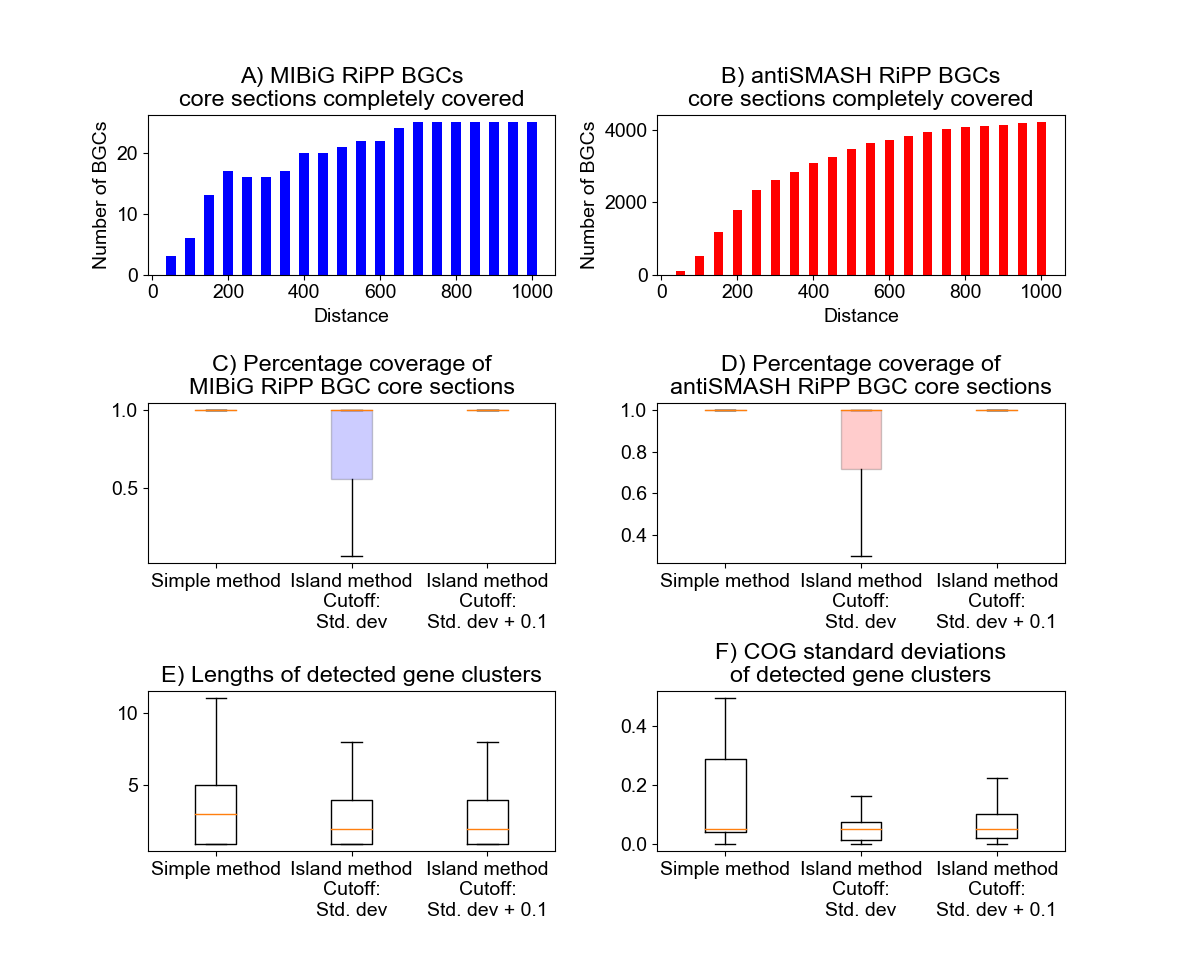

Supplement: S7 Fig — In the simple gene cluster formation method, genes are sequentially added as long as they are in the same strand orientation, within a certain distance. At a distance of 700 nucleotides, all MIBiG core gene sections are covered (A), as well as 91% (3947/4321) of antiSMASH core gene sections. (B). In the “island method,” genes are first fused into islands, which may be further fused if their average COG-scores are within a cutoff. Using just the standard deviation of the islands as a cutoff resulted in incomplete coverage of both the MIBiG and the antiSMASH core sections (C, D, middle boxes). Increasing the cutoff to the standard deviation plus 0.1 resulted in comparable coverage (C, D, right boxes) of these sections when compared to the simple method (C, D, left boxes). In addition, the overall gene cluster length (E) and variation of COG scores (F) within all formed gene clusters decreased. Numerical data of figures A, B, C, D, E, and F are available in S3 Data. BGC, biosynthetic gene cluster; COG, cluster of orthologous genes; MIBiG, Minimum Information about a Biosynthetic Gene cluster. (PNG) [file pbio.3001026.s007.png]

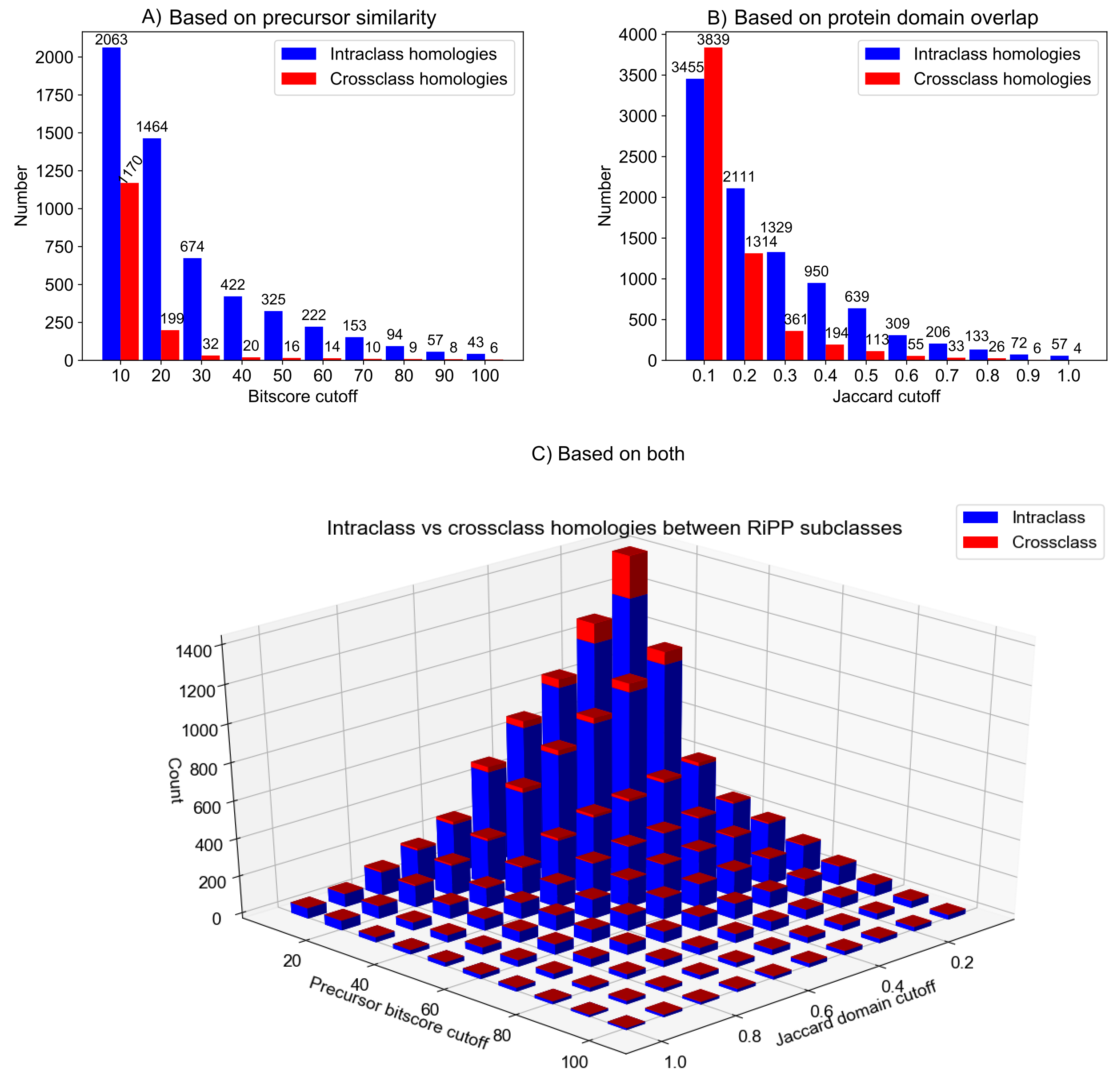

Supplement: S8 Fig — Starting at precursor similarity bitscore cutoffs of 20 and Jaccard scores of overlapping protein domains found in MIBiG RiPP BGCs of 0.4, the number of intraclass homologies is larger than the number of crossclass homologies. Combining the 2 methods greatly decreases the number of cross-class homologies found, proving it as an effective method to group RiPP BGCs of different subtypes. Numerical data of figures A, B, and C are available in S3 Data. BGC, biosynthetic gene cluster; MIBiG, Minimum Information about a Biosynthetic Gene cluster; RiPP, ribosomally synthesized and post-translationally modified peptide. (PNG) [file pbio.3001026.s008.png]

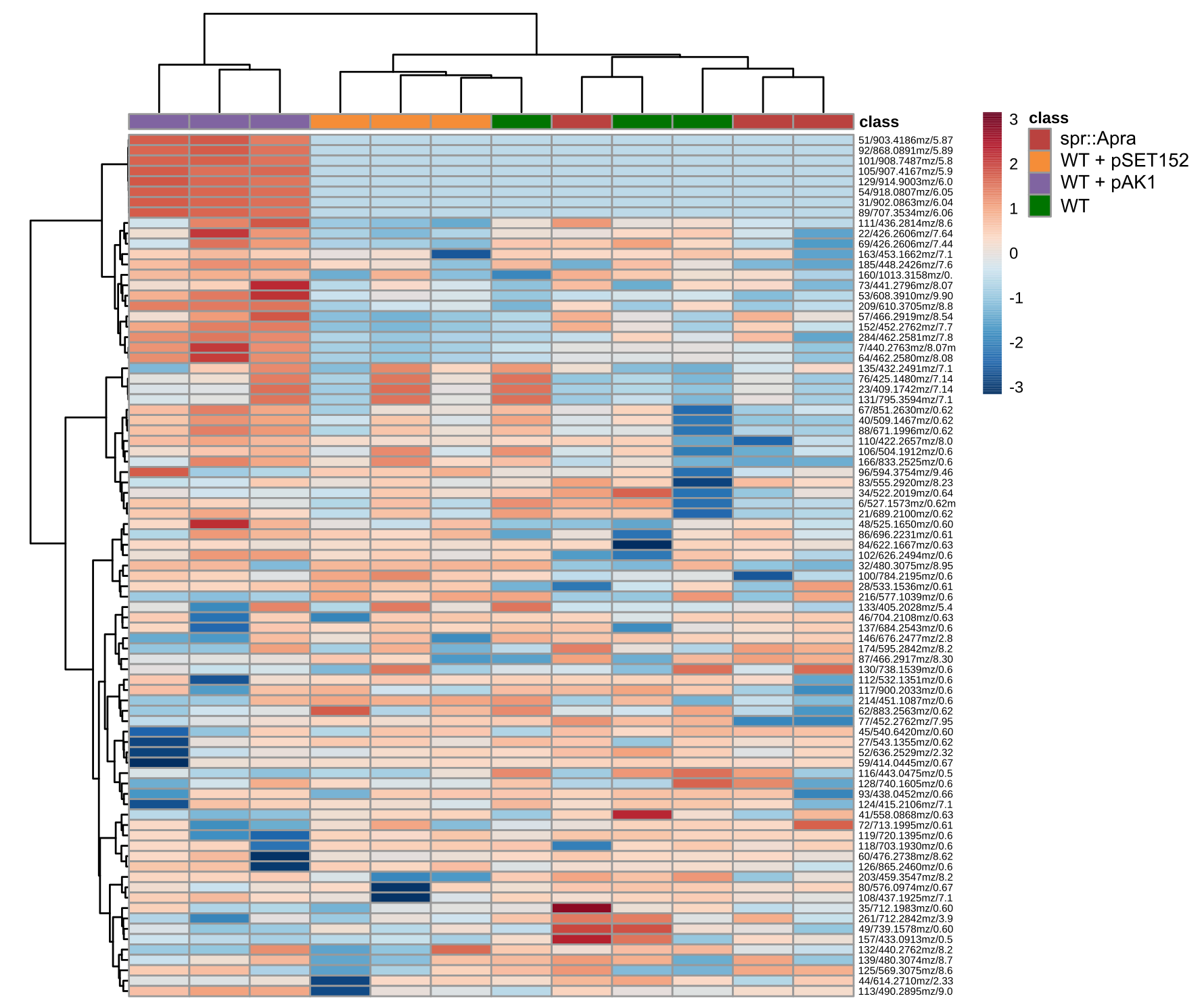

Supplement: S9 Fig — Color of the area indicates a log-fold increase. Numerical data are available in S3 Data. (PNG) [file pbio.3001026.s009.png]

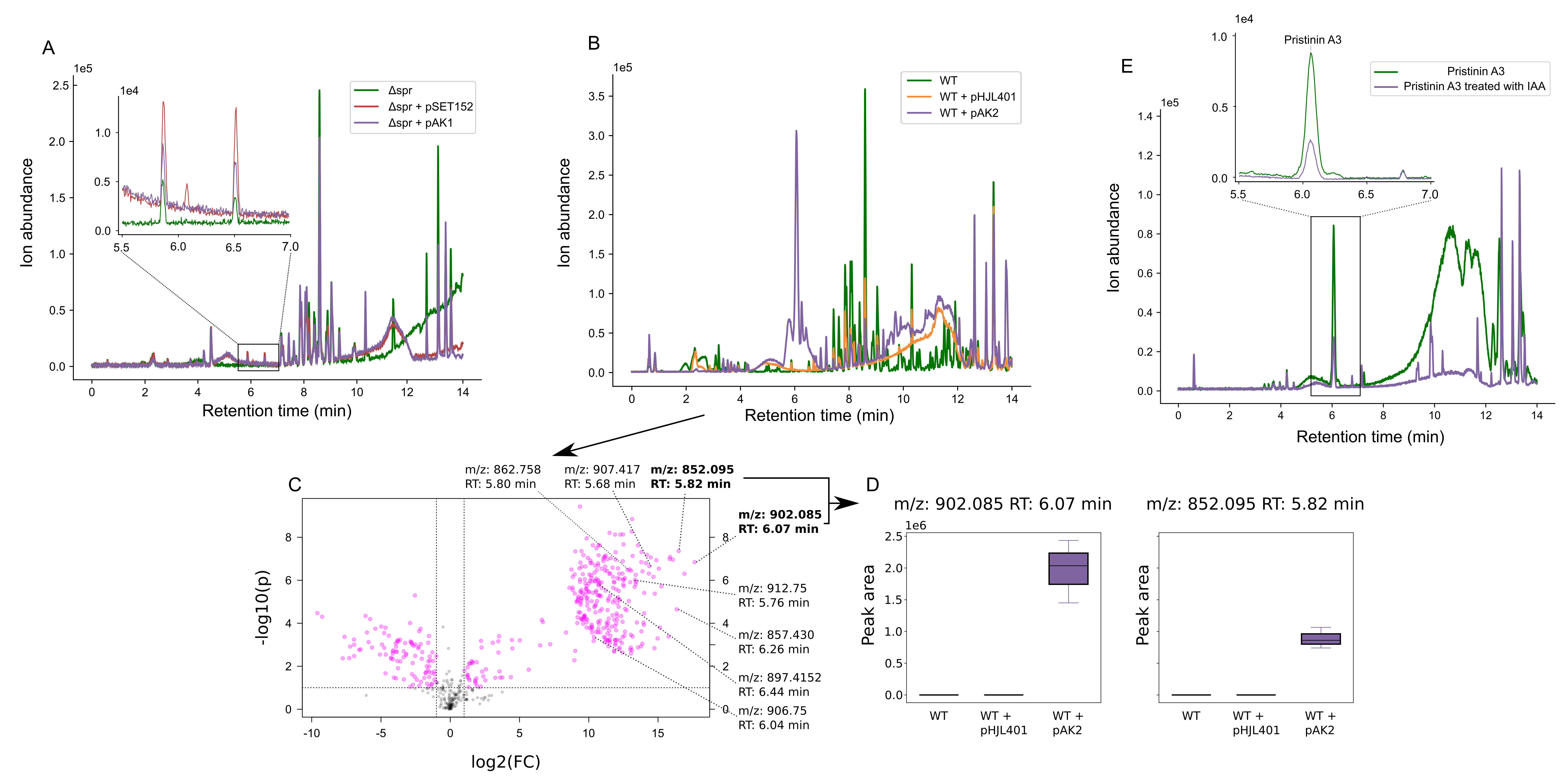

Supplement: S10 Fig — (A) Strains lacking the spr gene cluster are unable to produce the extracted products, even when transformed with pAK1. (B) Chromatogram of methanol extracts made from S. pristinaespiralis harboring no vector (WT), an empty pHJL401 vector (pHJL401), or pAK2 (pHJL401 with sprR behind pgap). A large peak can be seen in the extracts of strains harboring pAK2, not seen in extracts of the other strains. (C) Volcano plot comparing extracts of the strain containing pAK2 with the strain containing pHJL401. Peaks in pink had p-value ≥ 0.1 and a fold-change of ≥ 2. A large collection of peaks can be identified with log2(fold-change) ≥ 10. The 2 largest peaks (bold) corresponding to different monoisotopic masses could be related to the SprA2 and SprA3 precursors by MS/MS (S11 Fig). Many of the other masses eluted at comparable times and had masses that were close to the 2 major peaks, suggesting they were derived from them. Clear mass differences could be identified for some of the identified masses (S4B Data). Whether the largest peaks indeed correspond to the final product remains to be determined. (D) Extracted ion chromatograms of the 2 major peaks identified from the volcano plot. The 2 masses were only detected in the strain harboring pAK2. (E) Labeling experiments with IAA provide further support for the proposed structure of SprA3. (Purple) IAA covalently attaches to free sulfur groups of cysteines. However, the SprA3 peak was unaltered by IAA treatment, despite the presence of 3 cysteines in the peptide, strongly suggesting that these cysteines are not free. Numerical data of figures A, B, C, D, and E are available in S3 Data. IAA, iodoacetamide; MS/MS, tandem mass spectrometry; WT, wild-type. (PNG) [file pbio.3001026.s010.png]

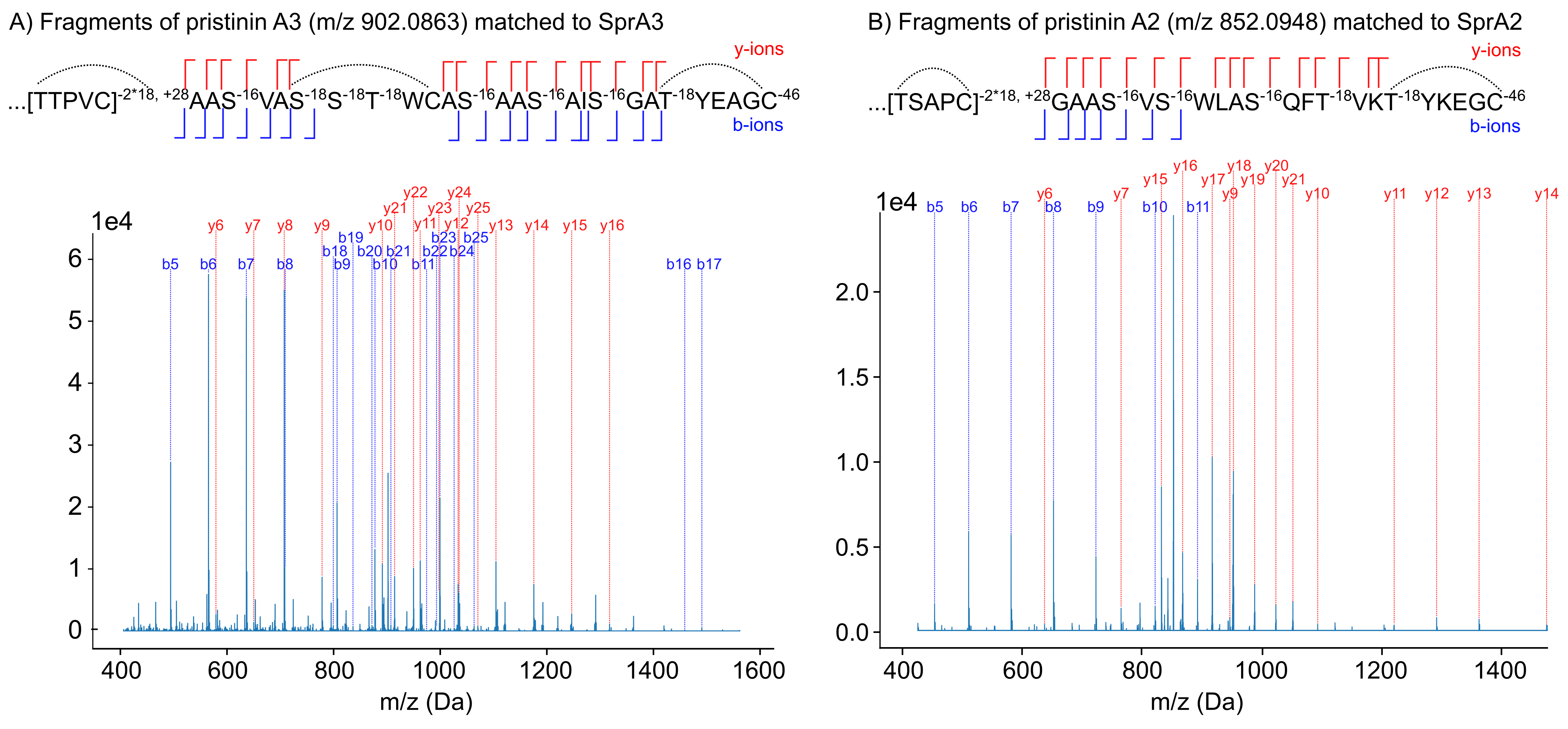

Supplement: S11 Fig — A full list of the modifications applied can be found in S4C and S4D Data. Numerical data of the chromatogram are available in S3 Data. (PNG) [file pbio.3001026.s011.png]

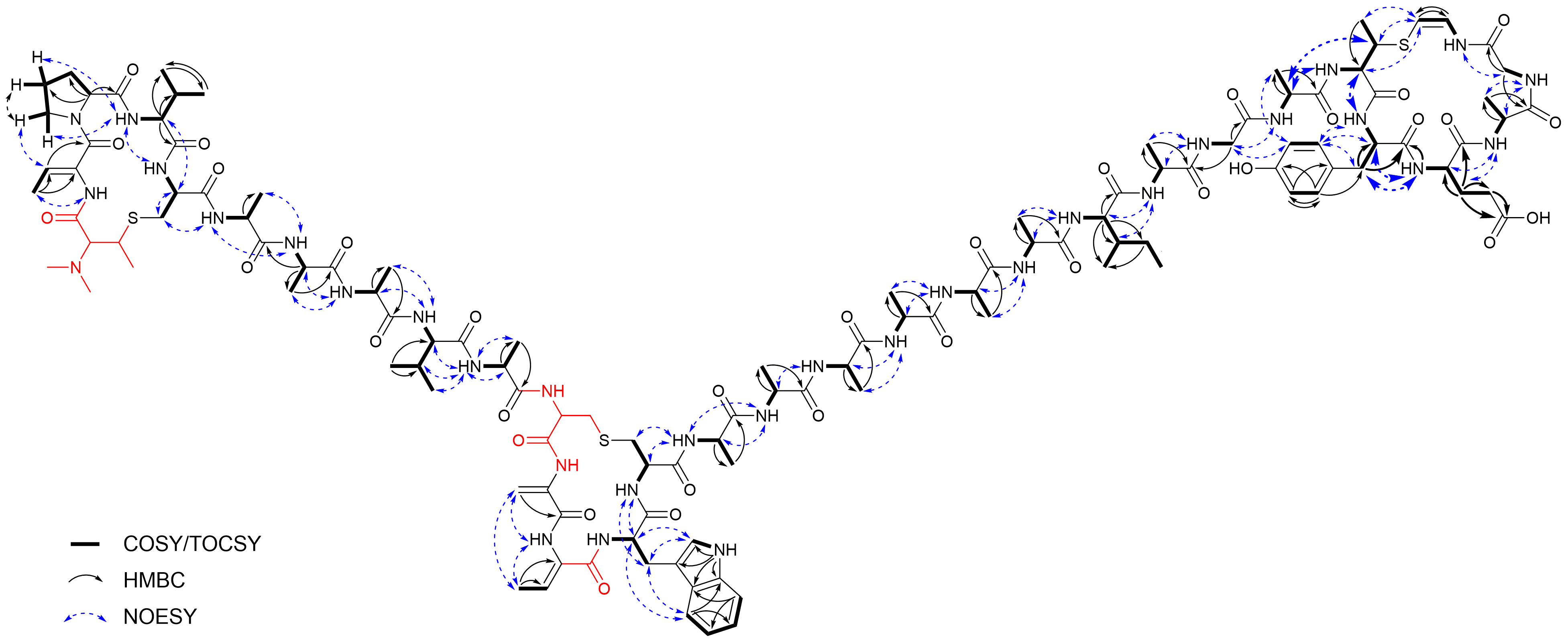

Supplement: S12 Fig — No clear correlations could be observed for the red parts of the structure, which were confirmed through other techniques. Bold arrows are for correlations which were better observed in CD3CN:H2O 9:1. (TIF) [file pbio.3001026.s012.tif]

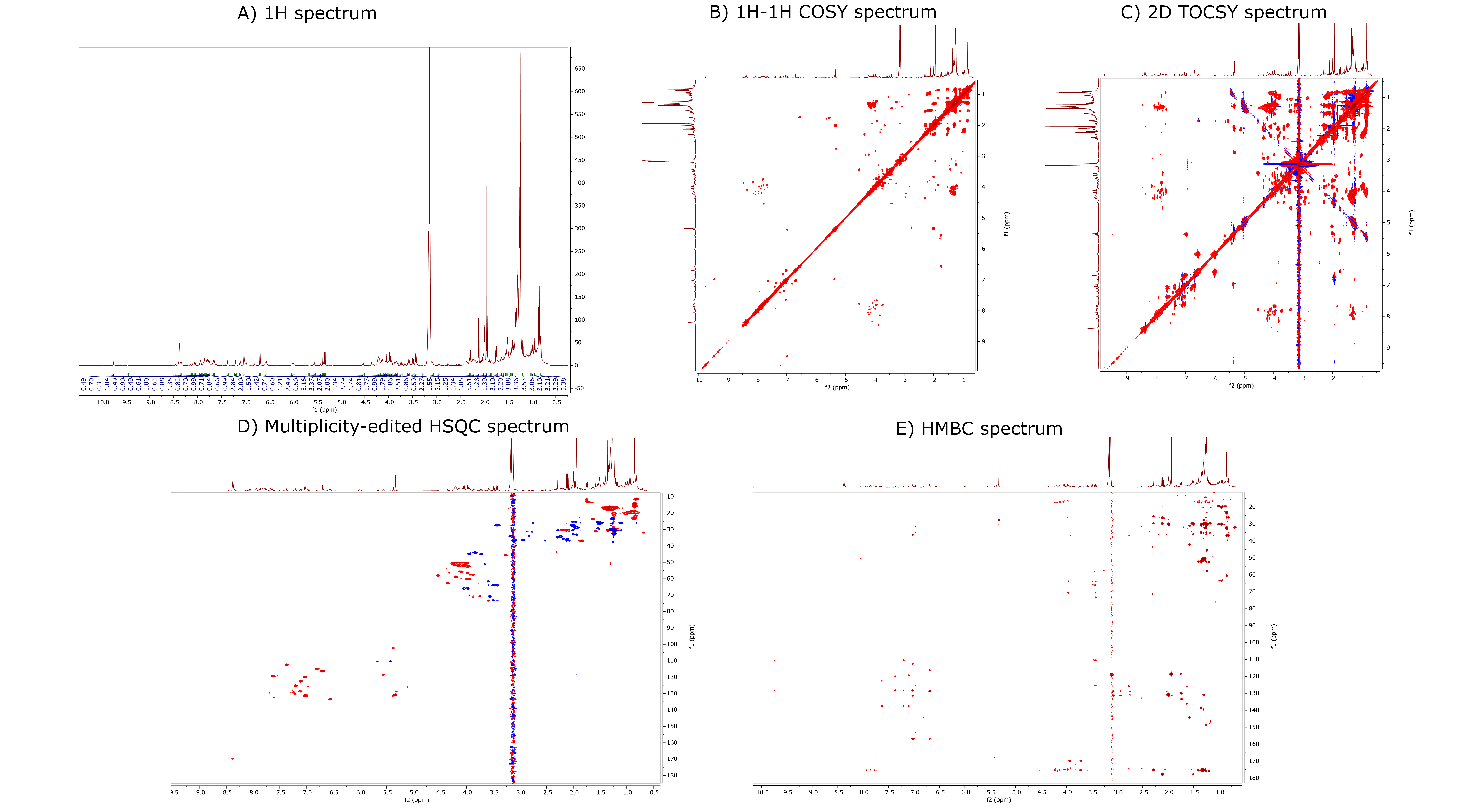

Supplement: S14 Fig — (A) 1H NMR spectrum with water suppression. (B) 1H–1H COSY spectrum. (C) 2D TOCSY spectrum. (D) Multiplicity-edited HSQC spectrum. (E) HMBC spectrum. (PNG) [file pbio.3001026.s014.png]

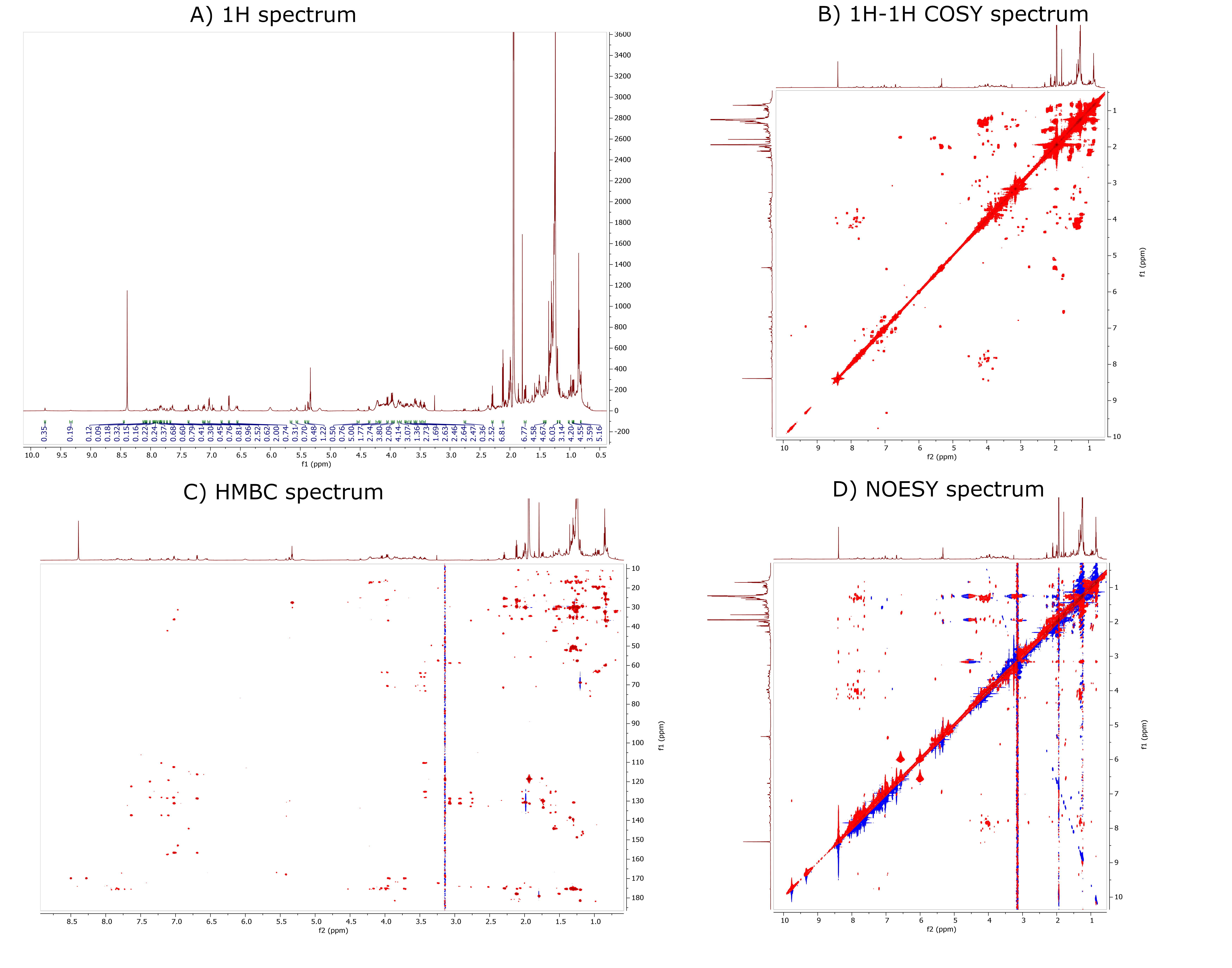

Supplement: S15 Fig — (A) 1H NMR spectrum. (B) 1H–1H COSY spectrum. (C) NOESY spectrum. (D) HMBC spectrum. (PNG) [file pbio.3001026.s015.png]

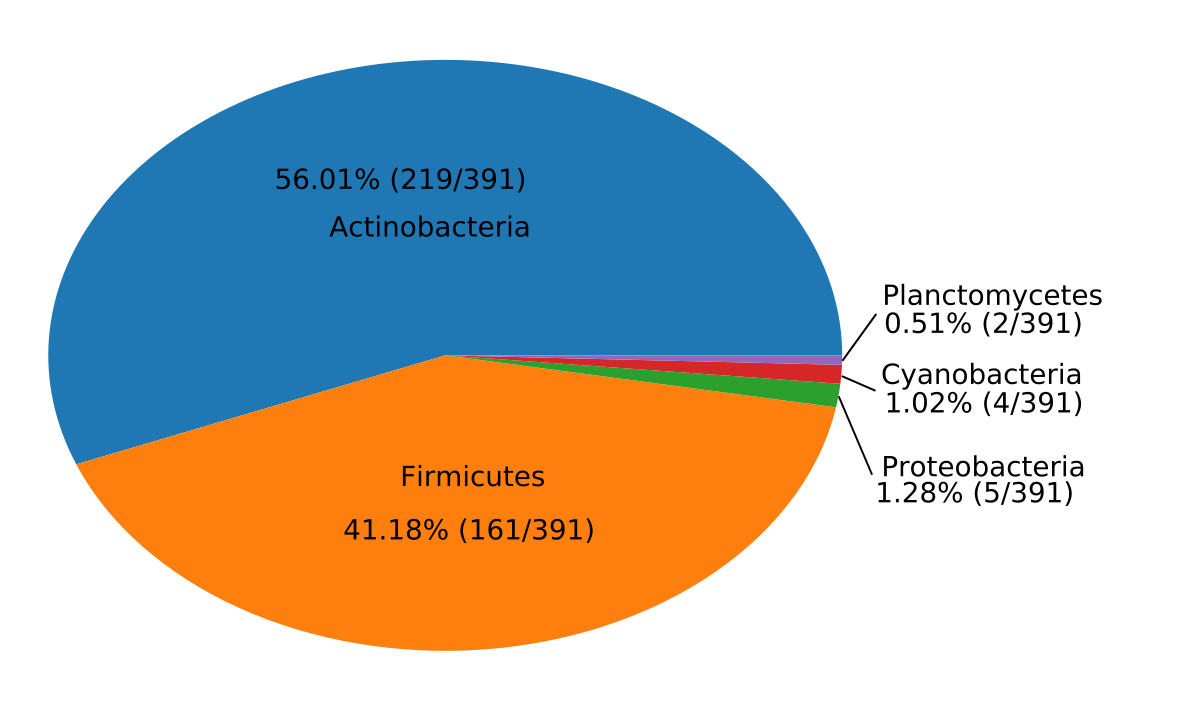

Supplement: S16 Fig — Most homologs were found in Actinobacteria and Firmicutes, although a few additional candidates were found in Proteobacteria, Cyanobacteria, and Planctomycetes. (PNG) [file pbio.3001026.s016.png]

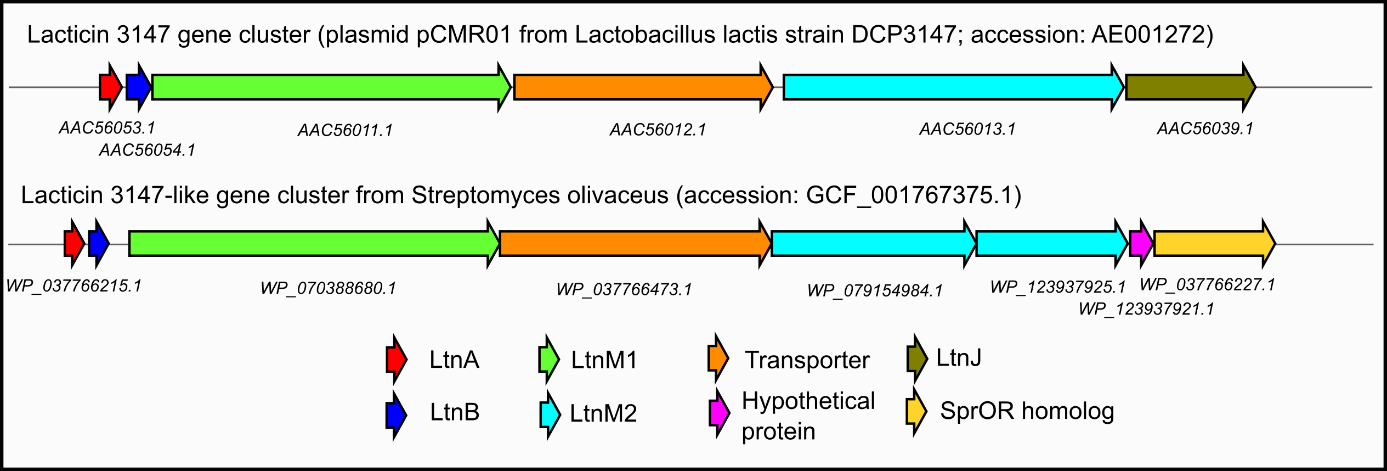

Supplement: S17 Fig — Genes encoding both precursors, both LanM-like modifying enzymes and the transporter are well conserved between the clusters. The gene encoding LtnJ, however, responsible for the reduction in the conversion to alanine and butyric acid, was not conserved. Instead, a homolog to sprOR was found, suggesting it may carry out a similar function. (PNG) [file pbio.3001026.s017.png]
