# Supplementary material for: Expansion of RiPP biosynthetic space through integration of pan-genomics and machine learning uncovers a novel class of lanthipeptides
Source: PLoS Biol. 2020 Dec 22;18(12):e3001026. doi: 10.1371/journal.pbio.3001026 (PMC7794033; doi:10.1371/journal.pbio.3001026)
Supplement: S1 Text — Precursors were aligned with MUSCLE [101] and visualized with BoxShade. (DOCX) [file pbio.3001026.s027.docx]

prod_559746 1 ---MHTM-TETDLLSGYTAYTTAEELDQFDGKAAPAATTPVLAPILI-----RASIIAARSSQQCA----AGIAAAGGGIWRTIRKVC
prod_4312120 1 ---MQNV-TEKDLFDGYTAYTSAEELGLHDGATAGPAFSPTV-PWAI-----QATVISARSSQACA----AALGSLAA---KTVEKKC
prod_4312121 1 ---MQNV-TEKDLFDGYTAYTSAEELGLHDGATAGPAFSPTV-PWAI-----QATVISARSSQACA----AALGSLAA---KTVEKKC
prod_9638834 1 ---MQNV-TEQDLFDGYTAYTSAEELGLHDGKDAAPAFSPTI-PWAI-----RATIISARSSQQCA----AALGSLAA---KTVENKC
prod_1888002 1 ---MQNV-TEKDLFDGYTAYTSAEELGLHDGQEAAPAFSPTI-PWAI-----RATIITARSSQQCA----AALGSLAA---KTVENKC
prod_1892473 1 ---MQNV-TEKDLFDGYTAYTSAEELGLHDGQEAAPAFSPTI-PWAI-----RATIITARSSQQCA----AALGSLAA---KTVENKC
prod_1898975 1 ---MQNV-TEKDLFDGYTAYTSAEELGLHDGQEAAPAFSPTI-PWAI-----RATIITARSSQQCA----AALGSLAA---KTVENKC
prod_2702012 1 ---MQNV-TEKDLFDGYTAYTSAEELGLHDGQEAAPAFSPTI-PWAI-----RATIITARSSQQCA----AALGSLAA---KTVENKC
prod_4125916 1 ---MQNV-TEKDLFDGYTAYTSAEELGLHDGQEAAPAFSPTI-PWAI-----RATIITARSSQQCA----AALGSLAA---KTVENKC
prod_4204099 1 ---MQNV-TEKDLFDGYTAYTSAEELGLHDGQEAAPAFSPTI-PWAI-----RATIITARSSQQCA----AALGSLAA---KTVENKC
prod_5620390 1 ---MQNV-TEKDLFDGYTAYTSAEELGLHDGQEAAPAFSPTI-PWAI-----RATIITARSSQQCA----AALGSLAA---KTVENKC
prod_5701534 1 ---MQNV-TEKDLFDGYTAYTSAEELGLHDGQEAAPAFSPTI-PWAI-----RATIITARSSQQCA----AALGSLAA---KTVENKC
prod_5937191 1 ---MQNV-TEKDLFDGYTAYTSAEELGLHDGQEAAPAFSPTI-PWAI-----RATIITARSSQQCA----AALGSLAA---KTVENKC
prod_6249001 1 ---MQNV-TEKDLFDGYTAYTSAEELGLHDGQEAAPAFSPTI-PWAI-----RATIITARSSQQCA----AALGSLAA---KTVENKC
prod_6819619 1 ---MQNV-TEKDLFDGYTAYTSAEELGLHDGQEAAPAFSPTI-PWAI-----RATIITARSSQQCA----AALGSLAA---KTVENKC
prod_710895 1 ---MQNV-TEKDLFDGYTAYTSAEELGLHDGQEAAPAFSPTI-PWAI-----RATIITARSSQQCA----AALGSLAA---KTVENKC
prod_7443641 1 ---MQNV-TEKDLFDGYTAYTSAEELGLHDGQEAAPAFSPTI-PWAI-----RATIITARSSQQCA----AALGSLAA---KTVENKC
prod_7703323 1 ---MQNV-TEKDLFDGYTAYTSAEELGLHDGQEAAPAFSPTI-PWAI-----RATIITARSSQQCA----AALGSLAA---KTVENKC
prod_8242019 1 ---MQNV-TEKDLFDGYTAYTSAEELGLHDGQEAAPAFSPTI-PWAI-----RATIITARSSQQCA----AALGSLAA---KTVENKC
prod_8466597 1 ---MQNV-TEKDLFDGYTAYTSAEELGLHDGQEAAPAFSPTI-PWAI-----RATIITARSSQQCA----AALGSLAA---KTVENKC
prod_8698113 1 ---MQNV-TEKDLFDGYTAYTSAEELGLHDGQEAAPAFSPTI-PWAI-----RATIITARSSQQCA----AALGSLAA---KTVENKC
prod_8721923 1 ---MQNV-TEKDLFDGYTAYTSAEELGLHDGQEAAPAFSPTI-PWAI-----RATIITARSSQQCA----AALGSLAA---KTVENKC
prod_8902069 1 ---MQNV-TEKDLFDGYTAYTSAEELGLHDGQEAAPAFSPTI-PWAI-----RATIITARSSQQCA----AALGSLAA---KTVENKC
prod_9724047 1 ---MQNV-TEKDLFDGYTAYTSAEELGLHDGQEAAPAFSPTI-PWAI-----RATIITARSSQQCA----AALGSLAA---KTVENKC
prod_1317692 1 ---MQNV-TEKDLFDGYTAYTSAEELGLHDGKEAAPAFSPTI-PWAI-----RATIISARSSQQCA----AALGSLAA---KTVENKC
prod_3048582 1 ---MQNV-TEKDLFDGYTAYTSAEELGLHDGKEAAPAFSPTI-PWAI-----RATIITARSSQQCA----AALGSLAA---KTVENKC
prod_398364 1 ---MQSTQNEKDLFEGYTAYTSAEELGLYDGKDAAPAFSPTI-PWAI-----RATIITARSSQQCA----AAIGSLTA---KTIENKC
prod_7467458 1 ---MQSTQNEKDLFEGYTAYTSAEELGLYDGKDAAPAFSPTI-PWAI-----RATIITARSSQQCA----AAIGSLTA---KTIENKC
prod_5042396 1 ---MQNV-NEKDLFDGYTAYTSAEELGLYDGKDAAPAFSPTI-PWAI-----RAGLITARSSQQCA----AAIGSFTA---RTIESKC
prod_1644796 1 ---MNAS---AHLIAGYTAYTTAAEFDA-SITADAPAVTPAT-P--------SIALSIAESSYACG----AGVGASIG---ITFTKGC
prod_7595003 1 ---MNAS---AHLIAGYTAYTTAAEFDA-SITADAPAVTPAT-P--------SIALSIAESSYACG----AGVGASIG---ITFTKGC
prod_9224211 1 ---VNTT---ENLIAGYTAYTSAQEIEA-THAEEAPGATPSV---------LSFIATSGWA---CG----AGIGTSIG---VTAAKGC
prod_4694754 1 ---VNTT---DTLLAGYAAYTSADEIAA-AQDGGAPEISPVS----L-----SIAVSIAESSYACS----AGLSMSVG---VTVGKGC
prod_4694755 1 ---VNTT---DTLLAGYAAYTSADEIAA-AQDGGAPEISPVS----L-----SIAVSIAESSYACS----AGLSMSVG---VTVGKGC
prod_7200544 1 ---MNTS---DNLMAGYATYTSADEIAA-TLDGGAPEISPVS----L-----SIAVSITESSYACG----AGISLSVG---WTVGKGC
prod_9224208 1 ---MNTA---DQLMAGYAVYTTSDEIGA-GAAADAPAISPVS---IF-----SAASSVECAIFSAG----VVTSASAG---GTVAGNC
prod_4694758 1 ---MNTA---DQLIAGYTAYTDSAEIAA-DATAEAPAISPTT----------TITIVSVESVLASI----GASASFSAG--YTVSSGC
prod_7200547 1 ---MNNT---DQLIAGYTAYTDSAEIAA-DASAEAPAITPTT----------TITIVSVESAVFSI----GGAASFSAG--YTISSGC
prod_326225 1 --MSHDQNTLEELVTGYESYADADEIEV-DAVTGAPATTPFCGA--------AASFMLSYV---------------------TTNGPG
prod_326226 1 --MTNDQSTLEDLVTGYESYADADEIEV-DAVTGAPATTPFCGA--------VASFALSYV---------------------TTNGPG
prod_6174086 1 ---VKTQ----DLIAGYAAYVDVAELNV-SAASEAPATSPVCFAAATSSAACLAATSSGWCVAGAG----AGVGGGIA---QSVKHGC
prod_6174087 1 ---M----ELDEMISGYDTYVDVAELDV-SAQSEAPATSPTC-----------FIASVGLS--------------------YQITKDL
prod_9167739 1 ---MQNDIEIMELVGGFEAYTEAAELNM-EASVEAPAATPTA------------TIVYTKFS----------VASVT----LTAKKGC
prod_2743547 1 VQKNDTV-DIMELVGGFEAYAEAAELNF-EASADAPAITPTL-----------TTIAYTKVS----------VASVSA----SIKVGC
prod_5868070 1 VQNIENV-EIMELVGGFEAYAQAAELNF-EASADAPAITPTL-----------TTIAYTKVTVAGT----A----------ASIKWTC
prod_1221493 1 ---MDTH----ELIEGFDAYVEAEELNE-DAMVDAPATTVPC-----------------------------TVASF-----ATGYFSC
prod_3289459 1 ---MDTH----ELIEGFDAYVEAEELNE-DAMVDAPATTVPC-----------------------------TVASF-----ATGYFSC
prod_467494 1 ---MDTH----ELIEGFDAYVEAEELNE-DAMVDAPATTVPC-----------------------------TVASF-----ATGYFSC
prod_1100745 1 ---MEKATSIVELLSGYEAYSSVEEINL-SAASDAPATTWGCAA---------VSASISWM-----------SGQVVS---KTVDDGC
prod_2725163 1 ---MEKATSIVELLSGYEAYSSVEEINL-SAASDAPATTWGCAA---------VSASISWM-----------SGQVVS---KTVDDGC
prod_3616888 1 ---MEKATSIVELLSGYEAYSSVEEINL-SAASDAPATTWGCAA---------VSASISWM-----------SGQVVS---KTVDDGC
prod_5244387 1 ---MEKATSIVELLSGYEAYSSVEEINL-SAASDAPATTWGCAA---------VSASISWM-----------SGQVVS---KTVDDGC
prod_6023772 1 ---MEKATSIVELLSGYEAYSSVEEINL-SAASDAPATTWGCAA---------VSASISWM-----------SGQVVS---KTVDDGC
prod_6473304 1 ---MEKATSIVELLSGYEAYSSVEEINL-SAASDAPATTWGCAA---------VSASISWM-----------SGQVVS---KTVDDGC
prod_6857183 1 ---MEKATSIVELLSGYEAYSSVEEINL-SAASDAPATTWGCAA---------VSASISWM-----------SGQVVS---KTVDDGC
prod_8409183 1 ---MEKATSIVELLSGYEAYSSVEEINL-SAASDAPATTWGCAA---------VSASISWM-----------SGQVVS---KTVDDGC
prod_9246364 1 ---MEKATSIVELLSGYEAYSSVEEINL-SAASDAPATTWGCAA---------VSASISWM-----------SGQVVS---KTVDDGC
prod_9674514 1 ---MEKATSIVELLSGYEAYSSVEEINL-SAASDAPATTWGCAA---------VSASISWM-----------SGQVVS---KTVDDGC
prod_1949441 1 ---MEKATSIVELLSGYEAYSSAEEINL-SAATDAPATTWGCAA---------VSASVSWM-----------SGQVVS---KTVDDGC
prod_5478099 1 ---MEKATSIVELLSGYEAYSSAEEINL-SAATDAPATTWGCAA---------VSASVSWM-----------SGQVVS---KTVDDGC
prod_326224 1 ---MDNA-SMMDLVAGYNTYAEASELGI-QAVADAPATTPVCAATIA-----ASAVSSGWC---AS----AAASAAGG---ATYKLGC
sprA3 1 ---MQNNTEIMDLIANYDAYADVDELNV-TAAADAPATTPVCAA---------SVASSTW----CA----SAASAISG---ATYEAGC
prod_8036387 1 --MSNKSTVIADLVAGYDAYTEVDELNV-SAAAGAPATTWVC----------VSVVASRASSVKCGAWASAGASAVSG---ATYEITC
prod_2805062 1 --MDNKSTVITDLVAGYSTYTEAGELNV-SAAAGAPATTYIC----------ASVAISRVSSPRCA----ASASAVSG---ATYEWTC
prod_297319 1 --MDNKSTVITDLVAGYSTYTEAGELNV-SAAAGAPATTYIC----------ASVAISRVSSPRCA----ASASAVSG---ATYEWTC
prod_5421071 1 --MDNKSTVITDLVAGYSTYTEAGELNV-SAAAGAPATTYIC----------ASVAISRVSSPRCA----ASASAVSG---ATYEWTC
prod_5527162 1 --MDNKSTVITDLVAGYSTYTEAGELNV-SAAAGAPATTYIC----------ASVAISRVSSPRCA----ASASAVSG---ATYEWTC
prod_6582107 1 --MDNKSTVITDLVAGYSTYTEAGELNV-SAAAGAPATTYIC----------ASVAISRVSSPRCA----ASASAVSG---ATYEWTC
prod_8403914 1 --MDNKSTVITDLVAGYSTYTEAGELNV-SAAAGAPATTYIC----------ASVAISRVSSPRCA----ASASAVSG---ATYEWTC
prod_9151868 1 --MDNKSAVITDLVAGYSTYTEAGELNV-SAAAGAPATTYIC----------ASVAISRTSSVKCS----AAASAISG---ATYEWTC
prod_9381790 1 --MDNKSAVITDLVAGYSTYTEAGELNV-SAAAGAPATTYIC----------ASVAISRTSSVKCS----AAASAISG---ATYEWTC
prod_8036386 1 ---MKTT-TIMELAAGYDAYTGAEELEV-GATAEAPASTPLCAA--------AASAGVSWM-----------ASQFSA---RTISGGC
prod_9151867 1 ---MKTT-AIMELVAGYEVYADSAELQV-DATVNAPASTPAC-----------GAATVSWI-----------VSQFSA---KTVKDGC
prod_9381789 1 ---MKTT-AIMELVAGYEVYADSAELQV-DATVNAPASTPAC-----------GAATVSWI-----------VSQFSA---KTVKDGC
prod_2805061 1 ---MKTT-AIMELVAGYEVYADSAELQV-DAAVDAPASTPAC-----------AAATLSWI-----------VSQFSG---KTVKDGC
prod_297320 1 ---MKTT-AIMELVAGYEVYADSAELQV-DAAVDAPASTPAC-----------AAATLSWI-----------VSQFSG---KTVKDGC
prod_5421070 1 ---MKTT-AIMELVAGYEVYADSAELQV-DAAVDAPASTPAC-----------AAATLSWI-----------VSQFSG---KTVKDGC
prod_5527161 1 ---MKTT-AIMELVAGYEVYADSAELQV-DAAVDAPASTPAC-----------AAATLSWI-----------VSQFSG---KTVKDGC
prod_6582106 1 ---MKTT-AIMELVAGYEVYADSAELQV-DAAVDAPASTPAC-----------AAATLSWI-----------VSQFSG---KTVKDGC
prod_8403913 1 ---MKTT-AIMELVAGYEVYADSAELQV-DATVDAPASTPAC-----------AAATLSWI-----------VSQFSG---KTVKDGC
sprA1 1 MADLQQTGSISELVAGYDTYSEAGELVA-EAAADAPASTPTC-----------AAATISWL-----------GSQLTV---KTYKEGC

sprA2 1 ---MDKTGAITELIEGYDSYSDAEELNS-TAAAEAPATSAPC-----------GAASVSWL-----------ASQFTV---KTYKEGC
