# Supplementary material for: Expansion of RiPP biosynthetic space through integration of pan-genomics and machine learning uncovers a novel class of lanthipeptides
Source: PLoS Biol. 2020 Dec 22;18(12):e3001026. doi: 10.1371/journal.pbio.3001026 (PMC7794033; doi:10.1371/journal.pbio.3001026)
Supplement: Attachment — Submitted filename: Reply_to_Reviewers.docx [file pbio.3001026.s028.docx]

Dear Dr Roberts, Dear reviewers,

We thank you for the constructive comments that helped to greatly improve this manuscript. During the past months, we have addressed all the points raised, and specific responses can be found below. First of all, we have performed several comparisons of decRiPPter’s performance with RODEO, NeuRiPP and DeepRiPP, to further validate our approach and contextualize it in the light of other tools, and have performed new analyses of putative precursor peptide-encoding open reading frames from genomic regions annotated as intergenic. Second, we have been able to almost fully elucidate the structure of the RiPP product of the SprA3 precursor based on a combination of NMR spectroscopy, MS/MS analysis, and acid hydrolysis. This was a major undertaking, given that one hundred 20 ml cultures had to be combined to obtain roughly 1 mg of product (the genes were not expressed in larger cultures), but we are very happy that we were able to add this important piece of scientific work to the manuscript, which now makes clear that the newly identified RiPP forms a new class of lanthipeptides together with the recently published cacaoidin peptide (which is now also clearly referred to in the text).

Below, please find a point-by-point response to all points raised by the reviewers.

Reviewer #1:

In this manuscript, the authors present a very well-crafted and particularly useful bioinformatics tool to identify a class of biosynthetic genes that - so far - proved difficult to identify in bacterial genomes. This tool, DecRiPPter, allows to perform genome mining in order to pinpoint ribosomally synthesized and post-translationally modified peptides (RiPPs). Whereas biosynthetic genes that are associated with e.g. polyketides or nonribosomal peptides can be identified using class-specific markers of highly conserved domains, this is virtually impossible for RiPPs, which lack these necessary markers. The authors tackle this difficult problem with a powerful machine learning and a pan-genomics approach rather than relying on known tailoring enzymes. This has the advantage that novel RiPPs can be found rather than 're-discovering' RiPPs that are merely modifications of known ones. This approach is followed by a second layer of core and accessory profiling at the BGC level and then a network analysis with antiSMASH results.

The bioinformatics approach is reasonable and it is tested on isolate genomes not only to find novel RiPPs but they also chemically characterize them to show that they belong to a novel class thus confirming its proposed utility.

Overall, this is a very elegant approach whose applicability and usefulness has been nicely demonstrated.

Some minor points:

1) The authors focused on bacteria of the genus streptomyces yet natural products researchers would certainly like to use this tool for other genera such as Pseudomonas. It would be highly appreciated if the authors could comment if this tool also works for these bacteria.

In general, the tool should be widely applicable for any bacterial genus, because the machine-learning algorithm for precursor peptide detection has been trained on a wide diversity of RiPP classes from diverse phyla, and because identification of enzyme-coding genes is independent of sequence similarity to known RiPP tailoring enzymes (thus avoiding a bias to highly-studied genera like *Streptomyces*). The main consideration in selecting a group of genomes is the COG-calculation. The genomes should be sufficiently closely related to one another so that they share a core genome, but should not be completely identical to each other. Any genera for which sufficient numbers of sufficiently diverse genomes are available would therefore qualify.

We expect that a suitable set of genomes can be selected for almost any genus for which at least a few dozen genomes are available, and have done preliminary work on ~200 different genera, using NCBI’s representative and reference genomes for each genus. The procedure worked for the majority (~90%), but failed for some of the larger genera. We expect that these issues can be resolved by tweaking the COG-parameters, or more cleverly selecting the genomes (e.g., splitting large genera into smaller subsets).

The manuscript has been updated on lines 370-374 to state that the method is not exclusive to streptomycetes.

2) The list of references is missing some bibliographical information. In particular references 21, 22, 25, 39, 54, 58, 60, 61, 71, 75 (e.g. journal names missing, etc.). Please update the bioRxiv citations 29, 30, and 76 which are all already published. Reference 16 and 34 is identical. Please re-check all references.

The reference library was indeed incomplete. It has been completely rebuilt and rechecked.

3) Lines 163-167: could be rephrased for better readability

4) Lines 344, 345, 385: please italicize 'S', 'Z', and 'm/z'

5) Line 378: please change 'likely cryptic' to 'likely silent'

6) Line 470: remove '.'

7) Line 362: The SI mentioned pAK3. Please include the plasmid name in manuscript.

8) Line 394-396: please include corresponding method description in the SI

The suggested text changes have been implemented and the typos have been corrected.

9)	There is a recent paper in Angew Chem (https://doi.org/10.1002/anie.202005187) that describes a novel RiPP - Cacaoidin. This compound seems to bear some resemblance to the RiPP described in this manuscript. Of course this does not influence the novelty of the authors' work yet it should be mentioned/addressed in the manuscript.

This paper was indeed published just around the time we submitted. The precursor peptide of cacaoidin showed no direct similarity to the *spr* precursor peptides, but the modifications are shared, and the modifying enzymes show similarity at the domain level. It is likely that the compounds belong to the same RiPP family. We have updated the manuscript to mention cacaoidin at lines 589-595 and in the section covered by lines 614-620.

Reviewer #2:

In this study, the authors focused on developing new bioinformatic methodology to find RiPP biosynthetic gene clusters (BGCs) that are not readily found by traditional means of looking for novel RiPP BGCs which mostly rely on knowledge of the PTM enzymes. The method is interesting as it could potentially discover new RiPP classes.

The method looks interesting to me, using a stepwise approach to finding such novel BGCs. The authors acknowledge that it could lead to many false positives and I agree, but if used as it is here, by growing up a potential candidate and looking for new compounds, I believe it could be valuable to prioritize candidates. The authors also acknowledge that true RiPP BGCs are also likely filtered out in their workflow, but at this stage I think that is OK. I must say that I am not a specialist in bioinformatic tool development. Hence my comments are those of a potential would-be user.

The authors mention that the last step is dereplication compared to known RiPP BGCs. They mention they used MIBiG and antiSMASH to do so. It must be said that neither of these are comprehensive at present and that classes may well be missed (as also indicated by lines 262-33). Can the authors state which currently known RiPP classes are being dereplicated by the current strategy? Are the currently known ~40 RiPP classes all represented in MIBiG or are representative examples for all found by antiSMASH?

Due to the timeline of our project, antiSMASH 4.0 and MIBiG 1.4 were used. These two were indeed not comprehensive, and some newer RiPP classes are likely not dereplicated. In particular, classes for which no antiSMASH detection rules have yet been established, can only be dereplicated by the MIBiG database. A few examples of these have shown up in our results, like the *tfuA-­*related BGCs. However, these are relatively rare, and the majority of filtered out RiPP BGCs were those belonging to well-established and widespread classes, like lanthipeptides and lasso peptides.

The most recent version of decRiPPter uses antiSMASH 5, and an update to the most recent version of MIBiG is pending.

The authors mention in the introduction that their method can potentially find clusters not found when using known PTM enzymes as queries using existing methods. But it must be said that the compound they describe could probably have been found using either the known family of Cy decarboxylases or the combination of SprH3/PT (which had already been suggested as the likely dehydratases for thioviridamide and linaridin biosynthesis). Hence, while I recognize that there are still many other candidate gene cluster families that the authors have not characterized, I think it should be mentioned that previous methodology (e.g. RODEO and perhaps DEEPRiPP) could have worked to find these class V lanthipeptides when applying the correct query. I think the authors should at least comment on this. I don't think that diminishes the value of this study, but it would place it better in context of other studies.

It is true that the gene cluster could have been identified using RODEO with these specific genes as queries; we mention this now on lines 426-427. In general, though, the difficulty in using RODEO or RiPPer is the selection of the right query enzyme, which requires expert knowledge. The Cys decarboxylase could indeed be used as a query, although as mentioned in the manuscript, it is also present in a number of non-RiPP BGCs and primary metabolic pathways, and may therefore not be as specific. DecRiPPter is the first unsupervised method to identify this RiPP BGC, and also identifies several other candidate RiPP families, which do not encode any RiPP marker.

As for the fact that the combination of the SprH3/PT has been suggested as likely dehydratases, we understand this situation to be slightly more complicated than the reviewer suggests here. For linaridin, there are two proteins which have been proposed as candidates for dehydration, called CypH and CypL. Which one is truly responsible is still unclear (see Ma, S. and Q. Zhang, Nat Prod Reports, 2020). These proteins are not similar, however, to any protein encoded in the thioviridamide-like BGCs, or to any encoded in the *spr* BGC.

As for the enzymes involved in the dehydration of thioviridamide-like compounds, these have to the best of our knowledge not been identified yet. The fact that two of the encoded proteins from the thioviridamide BGC show similarity to Spr3 and SprPT seems a promising indication that these proteins are involved in the relevant step, but we have seen no evidence in literature to confirm this. Indeed, the lack of any genes for known dehydration enzymes had been noted before (see Kudo, K. *et al*, ACS Chem Bio 2019, 14(6), pp. 1135-1140)

If we are mistaken in this, and the suggestion has been made before that the products of the *sprH3/PT* homologs are responsible for dehydration in thioviridamide-like compounds, we are of course happy to correct this, and add the relevant reference.

Another prior approach that deserves mentioning in the introduction is looking

for precursor genes near genes for AMC transporters (PLoS One. 2014;9(3):e91352. doi: 10.1371/journal.pone.0091352.). Although that study focused on lanthipeptides, and the current work obviously is much more comprehensive, I think it is a strategy that can be run agnostic of RiPP class.

The paper suggested here indeed shows the potential of using these transporters as a RiPP bait independent of specific RiPP classes. This is now mentioned in the introduction (lines 93-94).

Using their new platform, the authors discovered a new lanthipeptide and link it to a BGC using genetic studies that are well executed and quite convincing. Because the BGC does not contain the typical class I-IV lanthipeptide biosynthetic genes, the authors suggest this is a new class (class V to follow previous nomenclature first introduced by Sahl, although the current authors use "type" V in this paper; I recommend to be consistent and call them class V). This is exciting and interesting. The authors do not comment on a very recent paper that came to the same conclusions based on an activity-based fractionation approach that resulted in a new lanthipeptide (although the authors of that paper did not call the new group class V, it is clear its BGC has all the hallmarks of the current BGC and the structure of the two compounds are related). While I recognize that the current work was ongoing when that paper appeared, I still think the authors should discuss this other

paper and refer to it (Angew Chem Int Ed Engl. 2020 doi: 10.1002/anie.202005187.).

As mentioned above, this paper was published just before our submission, in fact on the day we planned to submit our paper. We therefore chose not to change the paper at that stage and submit to PLoS Biology, because of its "anti-scooping" policy (https://journals.plos.org/plosbiology/article?id=10.1371/journal.pbio.2005203). The precursor peptide of cacaoidin shows no direct similarity to the *spr* precursor peptides, but the modifications are shared, and the modifying enzymes show similarity at the domain level. It is likely that the compounds belong to the same RiPP class. We have updated the manuscript to mention cacaoidin at lines 589-595 and in the section covered by lines 614-620.

The suggestion that SprOR-like proteins may be responsible for formation of D-amino acids was also reached in another recent paper on lanthipeptide genome mining by isolating a two-component lanthipeptide with Ser-to-Ala conversions. Its BGC lacks LanJ but encodes an SprOR-like protein in its BGC, thus providing direct support for the hypothesis in Fig S18: BMC Genomics. 2020 21(1):387. doi: 10.1186/s12864-020-06785-7.

Thanks for pointing this out. We have missed this section in this work, but the enzymes do appear to be similar. A reference and mention of this work was added at lines 688-691.

Overall, I think this study is well suited for PLoS Biol after the authors address the comments above as well as specific comments below.

Specific comments:

Abstract "Most clinical drugs are based on microbial natural products". This statement is too general. It is true in some disease areas (e.g. infectious diseases) but is not correct for all clinical drugs.

We have reworked the first line of our abstract, no longer claiming that clinical drugs are mostly derived from natural products, but instead that natural products are a source for compounds that are potentially of clinical interest.

Are fluoroquinones (perhaps better fluoroquinolones?) really natural product derived? I think they are purely synthetic and not inspired or derived from natural products (their history goes back to an impurity in synthetic efforts to chloroquine synthesis).

Fluoroquinolones are indeed synthetic compounds, and their mention has been removed.

P3 "homologues of RiPP tailoring enzymes (RTEs) of interest". This nomenclature is not following the recommended nomenclature for RiPPs (ref 19). In the PKS/NRPS world the term tailoring is used for reactions that are compound specific and not class determining (they usually take place after the class determining reactions). In Arnison et al in 2013, the same use was recommended: tailoring reactions in RiPP biosynthesis are not class defining, but specific for a certain compound. Hence the definition here for RTEs is awkward as it goes against the community-agreed nomenclature. Class-defining or primary-modifications is what researchers in this area have used. I think it would be counterproductive to keep the nomenclature as it is introduced here (RTEs). Perhaps RPEs (RiPP primary-modification enzymes)?

The term RTE is somewhat ambiguous in our view. While we did not find any mention of the term in Arnison *et al*, 2013, as a term for an enzyme that is responsible for a modification that is not class-defining, we did find it mentioned as such in Funk & van der Donk*,* 2017 (Acc Chem Res). Additionally, at least two other papers use the term RTE, but then as a term for a more general modification enzyme (de los Santos, E.L.C, Sci Rep 2019 9(1), and Santos-Aberturas, J., Nucl Acids Res 2019 47(9)).

In general, we feel that the distinction between core and accessory modifications may become more and more ambiguous as more RiPPs are discovered. If our hypothesis for the proposed role of the SprH3 and SprPT gene pair is correct, they would be responsible for the formation of an AviCys in thioviridamides, considered an accessory modification, but also the formation of thioether bridges in lanthipeptides type V, considered a core modification. Whether to say these enzymes are RTEs or RPEs is a matter of debate, depending on the definition of the words, and until clear guidelines have been set we feel that using these terms will only cause confusion.

We have therefore chosen to drop the term RTE from our manuscript, and have instead used the more general term “modifying enzyme”.

P3, line 109 "by a previously unknown biosynthetic machinery". The machinery has been known in linaridin and thioviridamide biosynthesis (and in those cases proposed to be involved in dehydration). So, while it was not previously known for lanthipeptides, the machinery itself was certainly known (albeit not characterized, but it is also not characterized in this work).

As stated in more detail in the point addressed earlier, the thioviridamide BGCs and linaridin BGCs do not share a gene that could be involved in dehydration and/or cyclization. There has indeed been a suggestion of an enzyme that dehydrates linaridins, but as homologs of these enzymes are not encoded by the *spr* BGC, it is not relevant to our manuscript. As far as we know, there has been no suggestion for the enzymes involved in the dehydration/cyclization of thioviridamides.

P4 "To make sure that this classifier could predict precursors independent of RiPP subclass, we trained it on all possible subsets of the positive training set in which one of the RiPP subclasses was entirely left out". It would be helpful to show/discuss which subclasses the authors are referring to here. Indeed, in 2013 ~ 20 classes of RiPPs were known as the authors mention but that number is almost doubled (perhaps more than doubled?) and the current wording does not make clear which classes were used.

The training data was extracted early in the process, from MIBiG. As such, many of the newer RiPP classes were not used as positive training data. A table listing the RiPP types used in the training data has been added to the manuscript (S3 Table), and the training data is freely available as supplemental data. We expect a future iteration of the SVM classifier to take into account more RiPP precursors, and as such, show improved prediction capabilities.

Line 140. Define COG acronym at first use (now defined in line 172)

COG is now defined at its first use.

Line 141. "are then analyzed" would be better as "were then analyzed" (the remainder of the paragraph is all in past tense).

The tense has been changed.

Line 175. Define BUSCO at first use.

BUSCO is now defined at its first use.

"Families in which more than half of the gene clusters overlapped with antiSMASH non-RiPP BGCs were discarded as well," The authors might consider including a sentence that this step may eliminate BGCs that produce hybrids of RiPP and non-RiPP pathways which have been reported (e.g. ref 23 and Nat Chem Biol. 2018 Jul;14(7):652-654. doi: 10.1038/s41589-018-0068-6.)

We have added mention of RiPP hybrids at lines 302-304. Given the rarity of these RiPP hybrids, we feel that this filtering step is justified.

Line 308. Replace "sulfide" with "thiol" (sulfide is S2-)

Line 386. "showed that the six of the corresponding compounds"

Line 451: For consistency perhaps replace lantibiotics (only used here) with lanthipeptides (used throughout the paper).

Line 454: LanKL is not correct. This should be LanL.

Line 470. "available. , strongly supporting"

The typos and inconsistencies mentioned above have all been corrected, with thanks.

Line 482: "which is often associated with small prepeptides such as methanobactins". Methanobactins are not precursor peptides. They are the final RiPP.

This line has been rewritten to use the correct nomenclature (now lines 647-648).

The referencing is incomplete or outdated in many instances. This must be fixed prior to publication.

Many references are missing page numbers.

Ref 29. This bioRxiv paper from 2018 has been published since. Please replace. Nucleic Acids Res. 2019 May 21;47(9):4624-4637. doi: 10.1093/nar/gkz192

Ref 30. This bioRxiv paper from 2019 has been published since. Please replace. Sci Rep . 2019 Sep 16;9(1):13406. doi: 10.1038/s41598-019-49764-z.

Ref 39, 60, 61, 71, 75 is incomplete (missing year, volume, page numbers)

Ref 53: Referencing is incomplete. there are three other groups that reported on bottromycin in the same year in addition to this work (some of the others submitted earlier if the authors are to select just one; I think they should all be referenced).

The referencing library has been completely rebuilt, and each reference has been rechecked. There have been additional references added from 2012 describing the work of the three different groups discovering that botrromycin is a RiPP.

Figure 2. Phsophatase

The typo has been fixed.

Table 1. sprPT1 is suggested to be involved in Cys decarboxylation (last column). Why? LanD enzymes (SprF) do this without needing a phosphotransferase.

This was a mistake on our part in terms of naming the proteins. There are two encoded proteins with a phosphotransferase domain, one of which is uniquely associated with the Cys decarboxylase in *Streptomyces.* These two proteins, i.e. the Cys decarboxylase and the first phosphotransferase, are sometimes found fused as a single protein, and we presume them to be functionally closely related. These proteins are now called SprF1 and SprF2, while the other protein with a phosphotransferase domain is called SprPT.

Reviewer #3:

The authors describe the development of an algorithm and the program decRiPPter that allows predicting the biosynthetic gene clusters of ribosomally synthesized and post-translationally modified peptides (RiPPs). In contrast to existing methods, their new algorithm was specifically designed to detect also novel RiPP classes. They applied this algorithm to Streptomyces genomes and detected 42 new candidate RiPPs. One of them in S. pristinaespiralis was characterized in more detail and resulted in the discovery the first member of a new family of lanthipeptides. The distribution of two core genes of this family, the sprPT/sprH3 gene pair was found in many BGCs with more or less homology of the general BGC architecture. The elucidation of a novel RiPP is definitely a great discovery, yet, the merit of this manuscript and the underlying work is to been seen more in the development of decRiPPter which has been made freely available and the discovery that there are many more RiPPs

than could have been previously identified by antiSMASH or other programs. The only point I would like to rise is that it would have been preferable if the program decRiPPter could have been made available as web-based tool just like antiSMASH which would increase the user friendliness and the acceptance.

This may be an important tool for many researchers interested in natural products and give rise to the discovery of many novel RiPPs and RiPP-families.

The manuscript is well written and the research of highest quality. I have thus no doubts, that this manuscript should be accepted for publication in PLOS Biology.

We thank the reviewer for the positive feedback. Unfortunately, due to the size of the analyses to be done in a single run, and the fact that a large group of genomes needs to be analysed at the same time, setting up a web-based tool is infeasible. We do make all precalculated results for *Streptomyces* available freely online at <https://decrippter.bioinformatics.nl/>, and provide the tool as open-source software for any researchers to run it on their own data as they wish.

Reviewer #4:

This manuscript represents a very ambitious effort in identifying new RiPP biosynthetic gene clusters. Here, the authors have developed a series of bioinformatic rules (associated with a new tool, decRiPPter) that potentially represent a real advance in how gene clusters are identified. The analysis of conserved orthologous genes (COGs) seems a really neat way of identifying possible secondary metabolite clusters (RiPPs or otherwise), and the rules the authors define to identify likely RiPP gene clusters seem logical for the majority of clusters and does yield some really interesting candidate RiPP clusters. Below I will highlight both positive and negative aspects of the manuscript. On balance, I believe this will necessitate significant revisions to the manuscript prior to publication.

1. There are some real innovations in how it identifies new clusters, and the use of publicly available datasets to validate and refine the cluster mining approach is done very well. The ambition here is to be praised too, given that the focus is on novel clusters rather than new versions of existing RiPP families.

2. The online data is really appreciated and is well presented. This provides a substantial dataset for other researchers to investigate. It would benefit from a few improvements and corrections. Firstly, it would be much easier to navigate if strain names were associated with entries and/or there was a connection in some way to the data presented in the manuscript. For example, it wasn't easy to connect the 42 RiPP families described in the paper to the online information. One apparent minor error is that the genes in the GenBank files provided online seem to be in the wrong frame (forward strand only) when opened in gene viewer software. Also, the filter function doesn't seem to work online.

We thank the reviewer for the suggestion on this part, and have reworked the output files to contain the names of the organisms rather than their accessions. We have also uploaded the set of RiPP families manually curated from the strictly filtered set for easier navigation.

We have tested the .gbk files in gene viewer software (SnapGene), but we did not see the genes being in the wrong frame. If the issue persists, please open a new issue on the GitHub page or contact us directly.

3. A major concern is the likelihood of false positives that arise. The manuscript partially addresses this, but should do more to highlight and analyze this possibility for any potential users. An analysis of the online "strict" mode dataset picks out a number of clusters that are unlikely to be RiPPs:

- Clusters containing several consecutive phage structural proteins: 4 different ones, associated to 6 different precursor groups (1211, 22163, 4804, 2244, 1821, 6306)

- Clusters including a high proportion of sugar metabolism protein domains: precursor groups 5606, 10155, 76415, 12804, 50706, 170, 11811, 7713, 73777, 84405, 19730, 64989, 83901, 27291, 3156, 27600, 18839, 62573, 74379 (and maybe some more). Some of them could be real RiPP clusters, but it is possible that many of them are not

- Clusters that look like textbook RiPPs, such as RBGC_497026 (belonging to the large precursor group 462), but further domain analysis shows that the precursor has a LysW domain, which is characteristic of a carrier protein involved in lysine biosynthesis (http://www.nature.com/articles/nchembio.198). Other proteins encoded in the precursor group 462 clusters have similarity to this lysine biosynthesis pathway.

It is possible that some of these are filtered in the analysis described in the manuscript but not in the online data, as line 267 mentions that LysW peptides were discarded, yet do appear as "true" precursors via the strict mode online. Therefore, either some inconsistencies in the online data need correcting, or the authors should attempt to devise ways to further validate their findings and/or discuss false positive likelihood in the text.

We understand the concerns raised by the reviewer. In general, we feel that any explorative study that is not limited by a few RiPP-specific markers is bound to yield a considerable number of false positives. This is inherent to any ‘high-novelty / low-confidence’ approach (Medema & Fischbach, *Nat Chem Biol* 2015). A similar theme was also seen in the paper describing ClusterFinder (see Cimermancic *et al*, Cell 2014). It is through the stepwise elimination of false positives that we aimed to increase the saturation of true positives in our dataset, until the dataset could be manually curated.

The strict dataset uploaded was before manual curation, and still contained many non-RiPP BGCs. Many of these look promising at first sight, but further inspection eliminated many of them. We upheld the following criteria as a guideline for manual curation (also found in the manuscript, S3 Protocol):

1)	If more than half of the genes appeared involved in a known metabolic pathway not related to RiPPs, the BGC was filtered. This includes many examples, like the reviewers noted, including

a. The LysW pathway

b. Sugar metabolism pathways

c. Cell wall modification pathways

d. DNA modification pathways

In contrast, if only a few links to a known pathway could be identified, it seemed more likely to us that the candidate RiPP BGC was evolved from that pathway, such as in the MauE and MauD-encoding BGCs.

2)	The predicted precursor itself was annotated as a biosynthetic enzyme (commonly ferredoxin, which is also rich in cysteine residues). This step is done automatically in the currently version of decRiPPter, available on GitHub.

3)	The precursor was extracted from an intergenic region, but there appeared little space for a promoter region preceding it. This step may be automated in a later stage.

4)	The BGC contained some abnormalities, such as extreme lengths for RiPP BGCs (> 50 genes) or large intergenic gaps, or large genes (> 10,000 aa)

We now also provide the dataset after curation online at <https://decrippter.bioinformatics.nl/>.

There will always be bias in the criteria used, but this is not an issue if the criteria are clearly defined. Given how diverse RiPP BGCs are, there are no perfect criteria. Nonetheless, we hope the reviewer agrees that our approach uncovered many interesting novel RiPP candidates, which can be used to further chart the RiPP chemical space. The community can now have fun finding out what the real gems are among the 42 new RiPP candidate clusters.

Conversely, are there clusters that were picked up in previous genome mining studies that are not identified by decRiPPter but might have been expected?

We have compared our BGCs with those of antiSMASH and to those of the genome mining studies done using RODEO. Compared to these methods, 78% and 84% were detected by decRiPPter, respectively. decRiPPter is dependent on a RiPP precursor being encoded on the same strand as the rest of the BGC. It seems likely to us that many BGCs that are missed by decRiPPter either have a precursor peptide not encoded on the same strand as the biosynthetic genes, or the precursor peptide itself is not detected.

We have furthermore tested decRiPPter’s SVM on all of the precursor peptides identified by RODEO, of which it detected 66.7%. Given the fact that we are comparing only a single SVM to a collection of dedicated, class-specific SVMs, we feel that this percentage is acceptable.

The text has been updated on lines 145-150 and 283-288, and supplemental tables detailing the overlap with RODEO’s precursors and BGCs have been added (S4 Table, S6 Table).

4. Related to point 3, some further validation of the precursor peptide identification should be carried out. Due to the diversity of precursor peptide sequences, it seems likely that there will be a mixture of false positives (e.g. the examples mentioned in point 3) as well as missed precursor peptides when using an SVM approach. This isn't necessarily a big problem as the study aims to highlight unusual clusters, so some mis-called precursors seem acceptable, but are there any further approaches the authors could take to try to determine whether a predicted precursor peptide gene is likely to be real or not? For example, how many are already annotated as genes? If not, why not? A comparison with other precursor peptide assessment tools seems relevant here (e.g. NeuRiPP and DeepRiPP).

We have further investigated the differences between intergenic (i.e. extracted from intergenic regions) and non-intergenic (i.e. prodigal-detected) precursor genes. The majority of candidates are from intergenic hits. When inspecting the GC-plots of samples of these genes, we noted that prodigal-detected hits generally had GC-patterns as would be expected (i.e. the third position having the highest GC-percentage). The majority (90-95%) of intergenic hits had GC-patterns that were not following this pattern, and we recognize that they are a source of many of the false positives. Still, because of the sheer number of intergenic ORFs analysed, if only 10% of all intergenic hits would be considered, they would still constitute 41,9% of all hits. We therefore expect a significant part of the true positives to be derived from intergenic hits.

A more conservative run could only consider already annotated genes, and neglect the precursor genes in intergenic regions. This is a tradeoff between coverage and specificity; it is well known that many precursor genes are not annotated by gene-finding algorithms, but seeing as they may be a major source of false positives, the results of a run could be made more specific by neglecting intergenic hits.

The manuscript has been updated on lines 247-255 with a description of these analyses. The results can be found in S9 Figure.

We have additionally annotated all small ORFs from the 1,295 *Streptomyces* genomes with DeepRiPP and NeuRiPP. The fact that a lot of hits were found appeared to be common to all three precursor classifiers. DeepRiPP detected the most precursor candidates (4.4 million), then decRiPPter (1.3 million), and NeuRiPP the least (~430,000). Surprisingly, only a small subset overlapped between all three methods (~11,000). These 11,000 are likely a high-confidence set of RiPP precursors. It remains to be investigated how many of these belong to known RiPP precursors, although it is possible that a combination of different RiPP classifiers can result in a higher confidence in the results, by designing an ensemble algorithm. However, given the different training sets of the tools it is currently impossible to accurately estimate and compare the accuracies of the three classifiers, so a community effort would be required to do this in the future, involving retraining of all three classifiers on defined training sets and benchmarking on defined test sets.

We also found that most of the candidates among the 42 proposed ones do not have their precursors detected by NeuRiPP or DeepRiPP. We have updated the manuscript on lines 238-247, and added a new supplemental figure comparing the results (S8 Figure).

5. The authors make a significant claim about the discovery of type V lanthipeptides, but this needs further experimental validation given that the molecule has not been properly characterized. The manuscript has a very lengthy section on the attempts to identify the product and then partially characterize it, but simply needs proper NMR analysis of the product. The proper chemical characterization of one or more molecules in a mining paper like this is consistent with comparable studies (e.g. PNAS, 2020, 117, 371; NCB, 2017, 13, 470). This is especially important as a new class is being proposed, which includes speculation on a new set of proteins involved in the formation of D-amino acids.

We are excited to say that after several weeks of extensive new MS and NMR studies, we have now succeeded in obtaining the required structural information from the small amount of peptide we were able to purify from the SprA3 RiPP product. As detailed now in the paper, we are virtually certain of the proposed structure. All but two distant residues have been ascertained by NMR to match the proposed structure through MS/MS. We have ascertained the proposed modifications, including the dehydrated serine and threonine residues, the AviMeCys group, and the conversion of serine to alanine residues. To confirm the thioether crosslinks in the center and N-terminal ring structures, we provide partial evidence through NMR, together with a combination of acid hydrolysis, MS/MS analysis, labelling experiments with IAA, and the underlying precursor peptide sequence. We have added a new table, Table 3, which details exactly what experimental evidence was obtained per structural feature for each of the data sources. The revised section describing the structural characterization is covered by lines 490-587.

6. In relation to the designation of type V lanthipeptides, the gene pair that are described (sprH3/sprPT) are also present in clusters for the thioviridamides, which are not lanthipeptides. A qualification of this "type V" designation is therefore necessary - is it possible that some molecules are cyclized like lanthipeptides while others are dehydrated (or epimerized, based on the hypothesis associated with SprOR)? Or do these genes do something completely different given the lack of experimental data?

We have rewritten parts of the manuscript to reinforce the idea that even if the gene products of *sprH3* and *sprPT* are involved in dehydration and cyclization, that does not make their presence indicative of a lanthipeptide class V per se. The manuscript is updated accordingly on lines 616-621 and 657-662.

7. This may reflect a lack of experience with the interface, but it was difficult to find the spr gene cluster via the online interface. A search for the Streptomyces pristinaespiralis genome provided this output, which doesn't seem to include the spr cluster:

The gene cluster itself was not present in the strictly filtered set, due to the fact that the nearby regulator was not annotated with any Pfam domain, despite the fact it showed clear similarities to known LuxR-family regulators. We prioritized this BGC early on, primarily because the strain that contained it is genetically tractable.

The *spr* BGC can be found in the mildly filtered output: <https://decrippter.bioinformatics.nl/decrippter_mild/Operons/Filtered/Streptomyces_RBGC_308916/Streptomyces_RBGC_308916.html>

8. The data presented for the characterization of the spr product could be improved in the main manuscript. For example, Figure 3C shows a total ion current but doesn't directly relate this to the masses described in the text, while the reader has to go deeply into the SI to see full chromatograms of the various mutants (regulator over-expressed, delta-spr etc). The volcano plot presented in Figure S13 looks impressive, but it is difficult to understand what is presented - which peaks represent the predicted RiPP, what are the other peaks, and what does gray and pink denote? Improved presentation of this data would help guide the reader to the conclusions about the RiPP product.

Figure 3 and Figure S14, containing the volcano plot, have both been updated, and additional descriptions have been added to their legends where applicable. We hope the updated views make the data more understandable.

Further minor comments/corrections:

Line 14: I believe fluoroquinones were not inspired by natural products and represent a truly synthetic class of antibiotic.

The mention of fluoroquinolone has been removed.

Lines 167-172: the explanation here could be rephrased and simplified for non-experts. Would a figure help?

The section has been reworded and explained in more detail, and a more clear reference to the supplemental figure is given (Figure S2).

Line 193: "Namely" should be substituted by "For example", as some RiPP classes produced by Streptomyces are not included in that list (e.g. pheganomycin).

The correction has been made as suggested.

Lines 338-339: the differences between the spr precursors could be better visualized (currently embedded within a much larger alignment in Figure S10).

This alignment has been added to Figure 3.

Line 377-378. the manuscript reads as if only one medium was tested for production. If so, the statement that the spr cluster is cryptic should be removed. Is there any evidence that the cluster is not expressed under many different conditions? If only one medium is tested, many gene clusters are likely to be "cryptic".

We updated the manuscript to clarify that it is silent under the conditions tested.

Line 498: some more context for LtnJ and CrnJ would be useful to guide the reader - what is their conserved domain and what pathways are they from?

We have updated the manuscript, also using the more modern terms LanjA and LanjB, and now explain to which enzymatic pathways the families belong.

Line 504: "thus candidates" corrected to "thus is a candidate"

Line 506: "Table S10" corrected to "Table S11"

Table 2 needs correcting in places, such as naming of SprPT1 (SprF1?) and SprPT2, and the domain of SprF1.

We have corrected the typos and the Table as suggested, with thanks.
