# Supplementary material for: Expansion of RiPP biosynthetic space through integration of pan-genomics and machine learning uncovers a novel class of lanthipeptides
Source: PLoS Biol. 2020 Dec 22;18(12):e3001026. doi: 10.1371/journal.pbio.3001026 (PMC7794033; doi:10.1371/journal.pbio.3001026)
Supplement: Attachment — Submitted filename: Response_Editor_Reviewers.docx [file pbio.3001026.s029.docx]

**Editor comments**

a) Please attend to the remaining requests from reviewer #4.

>>> This was done, please see below.

b) Regarding this reviewer's comments about supplementary files, on the one hand, we do like the ability to link to specific components of the supplement, but on the other hand we strongly agree with the reviewer's comment that these could be more helpfully organised. Specifically, we suggest:

  1. Some of the Supplementary Figures are small and thematically linked. These should be combined into a single multi-panel Figure. This is recommended.

  2. Many of the Supplementary Tables are very small, very simple in structure, and thematically linked. These should be combined on single spreadsheets or on multiple sheets in single files. This is very strongly recommended.

  3. Supplementary Protocols 1 and 2 (and probably 3) should be incorporated into the main manuscript as part of a formal Materials and Methods section. This is essential.

>>> These suggestions have been carried out.

c) Please attend to my Data Policy requests further down this email.

>>> Another supplementary dataset has been provided, which provides numerical data of all the graphs and boxplots. The request for numerical data for Figure 4 caused some confusion – if by this the Newick Tree file is meant, it has been provided. For the NMR data, unfortunately no appropriate database for natural product raw NMR data is available at this point. A new initiative (<http://www.npmrd-project.org/>) funded by the NIH is busy solving this, but unfortunately their submission system does not work yet. We plan to submit our data there as soon as their system is ready for it.

d) Please could you change the title to something more explicit, such as "decRIPPter: integration of machine learning and pan-genomics expands the biosynthetic landscape of the RiPP natural product class"?

>>> Thanks for the suggestion and we understand the desire to emphasize the new software. However, the work is not only the novel machine learning but we also uncover a completely new class of lantibiotics. We feel that putting the software name in the title (and even more so at the start) would put too much emphasis on the software alone. However, we felt that we could increase the overall impact by changing the title to: "Expansion of RiPP biosynthetic space through integration of pan-genomics and machine learning uncovers a novel class of lantibiotics". We hope you are as happy with the new title as we are.

**Reviewer comments**

The majority of referee comments have been thoroughly answered and suitably addressed within the revised manuscript. This represents a tool that could be really valuable, as well as the characterization of an interesting new RiPP natural product.  I believe the manuscript is very suitable for publication now, where there are simply a few minor typographical errors that need addressing (Line numbers relate to the "R2" PDF that was supplied). In my opinion, these edits do not need further review:

* This may be the journal style, but "S3 Figure A" (Line 227) and similar supporting information nomenclature sounds odd

>>> This nomenclature style is required by the journal.

* It seems appropriate to name compound 1.

>>> We fully agree. We have chosen the name **pristinin** for these compounds, referring to the producer *S. pristinaespiralis*. Therefore, compound 1 has now been named **pristinin A3**, since it was derived from the SprA3 precursor.

* Line 220: Space needed between sentences
* Line 276: there appears to be a missing number in "7.18 *10"
* Line 424: Cysteine should be lower case spelling
* Figure S12: The text for each row is almost unreadable. Advise making the rows larger or text slightly smaller
* Line 525: There is some detail that seems unnecessary, such as "Even though the NMR shimming was appropriate, as evidenced from the solvent peak".  The authors could simply say that NH peaks were not visible in DMSO, so CD3CN:H2O was used to obtain clear NH signals

* Line 696: Re-word: "tools aimed at novelty may discovery new natural products". Perhaps: "tools aimed at novelty may lead to the discovery of new natural products".

>>> The wording has been changed and the typos corrected.

There are also a couple of additional minor comments:
1.  It is understandable that full stereochemical assignments aren't provided for compound 1, but cacaoidin includes D-alanine so is it worth commenting on this possibility for compound 1? It looks like there are serine residues that are converted to alanine residues, which would enable this.

>>> We believe this highly plausible, given that any serine-to-alanine conversion in lanthipeptides of which the stereochemistry was resolved reported D-alanines. We discuss this possibility in lines 588-590.

2.  I'm unsure whether this is a journal decision or an author decision, but the organization of data for this re-submission made reviewing much more difficult than should be necessary. If data is presented like this for the published paper, it is likely that a reader will ignore a lot of great supporting data. The following files had to be downloaded and viewed individually (I thought I'd list them all to show how much this is). I think this is all relevant data that deserves to be associated with the publication, but a single supporting info file would be much easier to follow (text, protocols, tables and embedded figures alongside their figure legends), as would a single Excel spreadsheet with multiple sheets (or a limited number of Excel files with multiple sheets).

>>> Many of the figures and tables have now been fused, either in multi-panel figures or in Excel documents.
